# Variant Exported Blood-Stage Proteins Encoded by *Plasmodium* Multigene Families Are Expressed in Liver Stages Where They Are Exported into the Parasitophorous Vacuole

**DOI:** 10.1371/journal.ppat.1005917

**Published:** 2016-11-16

**Authors:** Aurélie Fougère, Andrew P. Jackson, Dafni Paraskevi Bechtsi, Joanna A. M. Braks, Takeshi Annoura, Jannik Fonager, Roberta Spaccapelo, Jai Ramesar, Séverine Chevalley-Maurel, Onny Klop, Annelies M. A. van der Laan, Hans J. Tanke, Clemens H. M. Kocken, Erica M. Pasini, Shahid M. Khan, Ulrike Böhme, Christiaan van Ooij, Thomas D. Otto, Chris J. Janse, Blandine Franke-Fayard

**Affiliations:** 1 Leiden Malaria Research Group, Parasitology, Center of infectious Diseases, Leiden University Medical Center (LUMC), Leiden, The Netherlands; 2 Department of Infection Biology, Institute of Infection and Global Health, University of Liverpool, Liverpool, UnitedKingdom; 3 Department of Department of Parasitology, National Institute of Infectious Diseases (NIID), Tokyo, Japan; 4 Department of Microbiological Diagnostics and Virology, Statens Serum Institute, Copenhagen, Denmark; 5 Department of Experimental Medicine, University of Perugia, Italy; 6 Department of Molecular Cell Biology, Leiden University Medical Center (LUMC), Leiden, The Netherlands; 7 Biomedical Primate Research Centre (BPRC), Rijswijk, The Netherlands; 8 Wellcome Trust Sanger Institute, Hinxton, Cambridge, UnitedKingdom; 9 The Francis Crick Institute, Mill Hill Laboratory, Mill Hill, London, UnitedKingdom; Faculdade de Medicina da Universidade de Lisboa, PORTUGAL

## Abstract

Many variant proteins encoded by *Plasmodium*-specific multigene families are exported into red blood cells (RBC). *P*. *falciparum*-specific variant proteins encoded by the *var*, *stevor* and *rifin* multigene families are exported onto the surface of infected red blood cells (iRBC) and mediate interactions between iRBC and host cells resulting in tissue sequestration and rosetting. However, the precise function of most other *Plasmodium* multigene families encoding exported proteins is unknown. To understand the role of RBC-exported proteins of rodent malaria parasites (RMP) we analysed the expression and cellular location by fluorescent-tagging of members of the *pir*, *fam-a* and *fam-b* multigene families. Furthermore, we performed phylogenetic analyses of the *fam-a* and *fam-b* multigene families, which indicate that both families have a history of functional differentiation unique to RMP. We demonstrate for all three families that expression of family members in iRBC is not mutually exclusive. Most tagged proteins were transported into the iRBC cytoplasm but not onto the iRBC plasma membrane, indicating that they are unlikely to play a direct role in iRBC-host cell interactions. Unexpectedly, most family members are also expressed during the liver stage, where they are transported into the parasitophorous vacuole. This suggests that these protein families promote parasite development in both the liver and blood, either by supporting parasite development within hepatocytes and erythrocytes and/or by manipulating the host immune response. Indeed, in the case of Fam-A, which have a steroidogenic acute regulatory-related lipid transfer (START) domain, we found that several family members can transfer phosphatidylcholine *in vitro*. These observations indicate that these proteins may transport (host) phosphatidylcholine for membrane synthesis. This is the first demonstration of a biological function of any exported variant protein family of rodent malaria parasites.

## Author Summary

Malaria-parasites invade and multiply in hepatocytes and erythrocytes. The human parasite *P*. *falciparum* transports proteins encoded by multigene families onto the surface of erythrocytes, mediating interactions between infected red blood cells (iRBCs) and other host-cells and are thought to play a key role in parasite survival during blood-stage development. The function of other exported *Plasmodium* protein families remains largely unknown. We provide novel insights into expression and cellular location of proteins encoded by three large multigene families of rodent malaria parasites (Fam-a, Fam-b and PIR). Multiple members of the same family are expressed in a single iRBC, unlike *P*. *falciparum* PfEMP1 proteins where individual iRBCs express only a single member. Most proteins we examined are located in the RBC cytoplasm and are not transported onto the iRBC surface membrane, indicating that these proteins are unlikely to mediate interactions between iRBCs and host-cells. Unexpectedly, liver stages also express many of these proteins, where they locate to the vacuole surrounding the parasite inside the hepatocyte. In support of a role of these proteins for parasite growth within their host cells we provide evidence that Fam-A proteins have a role in uptake and transport of (host) phosphatidylcholine for parasite-membrane synthesis.

## Introduction

Malaria parasites (*Plasmodium* spp.) invade both liver cells and red blood cells (RBC) in the vertebrate host. They actively remodel the infected RBC (iRBC) by exporting and trafficking various proteins to the RBC cytoplasm and the plasma membrane [1–7]. These *Plasmodium* proteins are involved in different processes ranging from uptake of nutrients, the formation of membranous structures, protein trafficking and mediating adherence of iRBC to host cell receptors [5]. A large proportion of these exported proteins are encoded by multi-copy gene families and for several of these families evidence exist for their involvement in antigenic variation and immune evasion [6, 8–15]. PfEMP1 proteins encoded by the *P*. *falciparum var* gene family are transported to the surface of iRBC, where they mediate interactions of iRBC with host cells. These proteins operate as ligands that bind to host cell receptors on the capillary endothelium resulting in iRBC tissue sequestration, which is believed to prevent iRBC being removed by the spleen [16–20]. Furthermore, the small variant proteins of the *P*. *falciparum stevor* and *rifin* multigene families are involved in iRBC interactions such as rosetting and iRBC tissue sequestration [21–24]. STEVOR-mediated rosetting provides a growth advantage by protecting merozoites from invasion-blocking antibodies [21]. Both sequestration and rosetting of iRBC have been linked to virulence of *P*. *falciparum* infections [6, 18, 19, 25–29]. Proteins encoded by the *P*. *falciparum stevor* and *rifin* multigene families are thought to be distantly related to the *pir* multigene family of rodent and other non-human primate and human malaria parasites [8, 10, 30] and it has been suggested that PIRs of these species also mediate iRBC rosetting as well as iRBC tissue sequestration [21, 31, 32]. However, the molecular determinants mediating the interactions between PIRs and host cells are unknown.

The established functions for several *P*. *falciparum* exported proteins of multigene families in interactions between iRBC and host cells (i.e sequestration) or between iRBC and other RBC (i.e rosetting) may indicate that these proteins have mainly or exclusively a role in promoting parasite survival during the erythrocytic part of the life cycle. Most studies on exported proteins, however, have been performed in *P*. *falciparum* and the functions of exported proteins encoded by multigene families of other *Plasmodium* species remain largely unknown. For example, rodent malaria parasites (RMP) have several multigene families that encode variant exported proteins whose functions are unclear [10, 33–35]. In addition to the *pir* multigene family, RMP contain two large gene families, *fam-a* and *fam-b*, that encode exported proteins [33, 35, 36]. The *fam-a* family is the only multigene family of exported proteins in which a structural domain has been identified that is known from other organisms. This domain has similarity to the steroidogenic acute regulatory-related lipid transfer (START) domain [33, 35], suggesting that these proteins may play a role in the transport of lipids. Different PIR members may fulfil distinct functions as is suggested by the discovery of structurally distinct phylogenetic clades of PIRs represented in multiple species, coupled with differential expression and cellular location of different PIR members during the erythrocytic cycle [8, 9, 32, 33, 35, 37, 38]. PIR proteins are also expressed in blood-stages of *Plasmodium* species that lack iRBC sequestration or rosetting and in non-sequestering blood-stages such as (young) trophozoites and gametocytes of *P*. *berghei* [36]. Moreover, many PIR proteins are not located at the RBC surface membrane [36], suggesting that these proteins have additional roles beyond the promotion of sequestration or rosetting through interactions between iRBC and host cells. Multiple distinct functions have also been proposed for the *P*. *falciparum stevor* multigene family [21, 39].

In this study we have performed phylogenetic analyses of the RMP *fam-a* and *fam-b* multigene families and analysed expression and cellular localization of members of the *pir*, *fam-a* and *fam-b* families throughout the complete life cycle of *P*. *berghei*, including mosquito and pre-erythrocytic stages. To perform the phylogenetic analyses, we first re-sequenced the *P*. *berghei* genome utilizing the Pacific Biosciences single-molecule real-time (SMRT) sequencing technology [40], resulting in improved annotation of the *fam-a*, *fam-b* and *pir* families. For all three families we found that multiple proteins of the same family were expressed simultaneously in a single parasite. Most proteins were localized in the iRBC cytoplasm and not transported onto the surface membrane, indicating that these members play no direct role in interactions between iRBC and host cells. Unexpectedly we found that members of all three families that were expressed in iRBC were also expressed in late liver-stages where they are located in the parasitophorous vacuole (PV). For two Fam-a proteins we provide evidence for a location at the PV membrane. Their expression in late liver-stages suggest that these proteins promote parasite development not only in the blood but also in the liver either by supporting parasite development in both the infected hepatocyte and RBC and/or through manipulation of the host immune response. In support of a role in parasite growth within the host cell, we found that several Fam-A members transfer phosphatidylcholine (PC) *in vitro*. Since host cell PC has been shown to be key for malaria liver stage development [41], these observations indicate that Fam-A proteins may mediate the transfer of PC from the host cell into the parasite for membrane synthesis. This is the first demonstration of a biological function of any exported variant protein family of rodent malaria parasites.

## Results

### Identification of new *P*. *berghei* fam-a, fam-b and pir genes by annotation of an improved genome sequence

Recently we have reported an improved annotation of the genomes of the RMP *P*. *berghei*, *P*. *yoelii* and *P*. *chabaudi*, permitting the exact determination of the number and genomic location of the *pir*, *fam-a* and *fam-b* family members in the latter two species [33]. However, the presence of large arrays of 2.3kb repeat sequences and low complexity regions inside the subtelomeric regions of *P*. *berghei* chromosomes has hampered correct annotation of *P*. *berghei* multigene family members. To further improve the *P*. *berghei* genome annotation we first assembled the *P*. *berghei* genome sequence based on new sequence information obtained with the Pacific Biosciences single-molecule real-time (SMRT) sequencing technology that successfully can resolve long repeat sequences [40]. This resulted in a core *P*. *berghei* genome without gaps but with a remaining 11 contigs that could not be attached due to the presence of repeat sequences such as the tandem 2.3 kb repeat sequences. A total of 34, 18 and 44 novel *fam*-a, *fam-b* and *pir* genes, respectively, were annotated (**S1 Table**) and these were included in the phylogenetic and expression analyses shown below. The new sequences, gene annotation and genomic location are published on the GeneDB website (www.GeneDb.org).

### Conserved, structural differences between members of the fam-a and fam-b gene families

**Fig 1:**
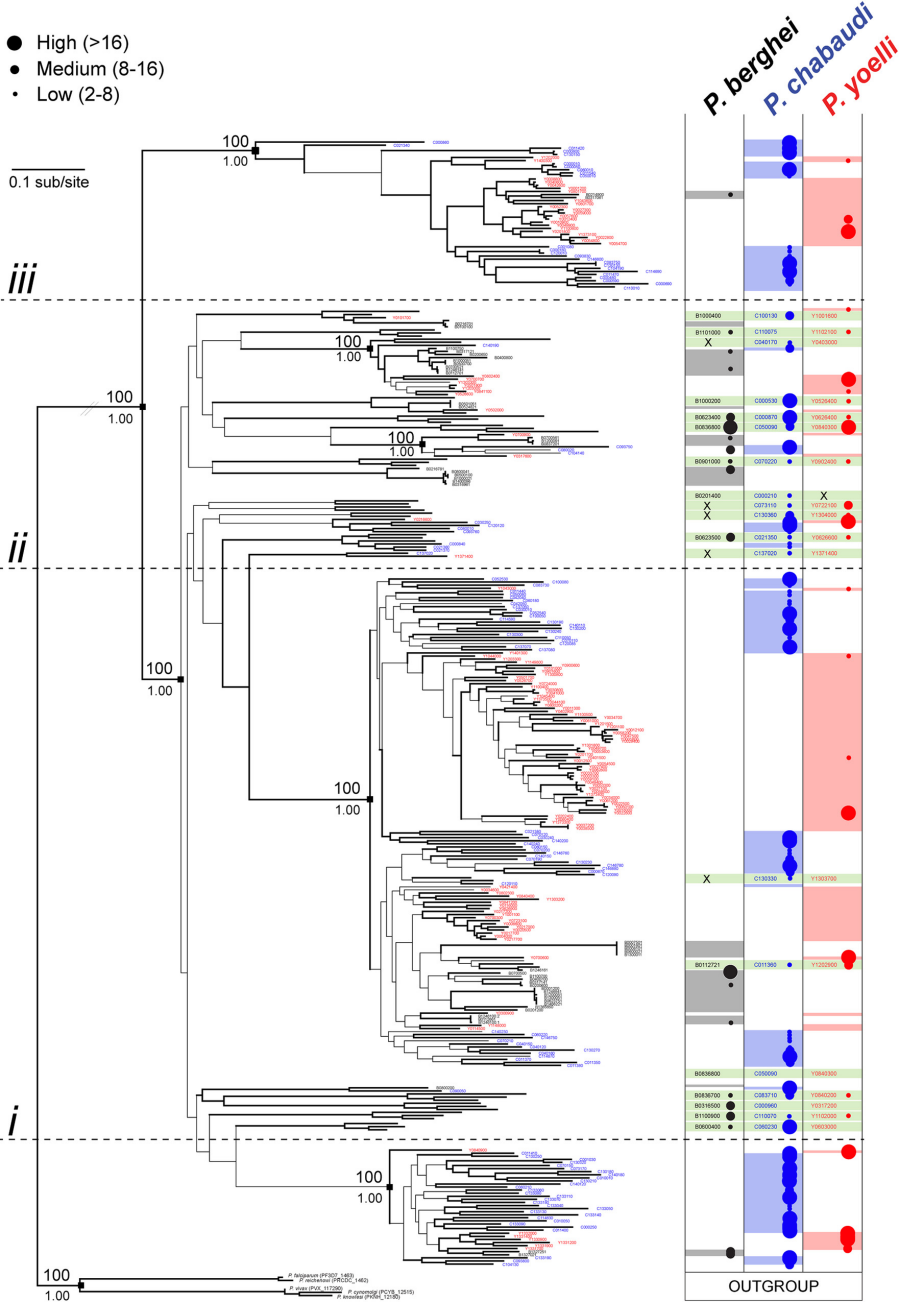
Maximum likelihood phylogeny of *fam-a* gene sequences from *Plasmodium* spp. The tree was estimated using RAxML and a GTR+Γ model. Branches subtended by nodes with >75 bootstrap support are shown in bold. Robust basal nodes are indicated by black squares with bootstrap proportions (above node) and Bayesian posterior probabilities (beneath node). At right, coloured blocks indicate the species to which a terminal node belongs. Clades of orthologs that display positional conservation are indicated with green blocks; where a sequence has been lost secondarily in a species is shown by an ‘X’. The tree is rooted using an out-group comprising single copy *fam-a* orthologs from primate *Plasmodium* species. The phylogeny is subdivided into four sections: genes located at the conserved, ‘ancestral’ locus on chromosome 13 (below line *i*); genes found at loci conserved across RMP species (between lines *i* and *ii*); and a robust clade of species-specific paralogs derived from a conserved locus on chromosome 6 or 13 (between lines *ii* and *iii*); a robust clade of species-specific paralogs derived from a conserved locus on chromosome 8 (above line *iii*).Transcription levels (shown as different coloured and sized circles) in blood stages are shown for individual genes based on RNAseq data (FPKM values) (from [33] and **S1** Table). Expression levels as shown by four different sized circles: Class 1 (smallest circle): 2-8x the threshold level; class 2: 8-16x the threshold; class 3 (largest circle): >16x the threshold.

A recent phylogenetic analysis of the *pir* family of all three RMP demonstrated the presence of robust clades, each characterized by distinct structural motifs and represented in multiple species, which may indicate a long-standing functional diversification among members of this family [33]. In order to determine whether the *fam-a* and *fam-b* families can be similarly differentiated into consistent and robust phylotypes, we performed a phylogenetic analysis of these families using approaches similar to those used to generate the RMP *pir* phylogeny. Most *fam-a* genes have a subtelomeric location but all three RMP have multiple copies that share a syntenic location in an internal region of chromosome 13 [33, 35]. The *fam-a* genes comprise of six exons and five introns and encode proteins of approximately 300 amino acids. RMP Fam-a proteins are characterized by the PYST-A domain and most members have a predicted signal peptide but lack a PEXEL and transmembrane domain (www.GeneDB.org; [33]). The maximum likelihood (ML) phylogeny of the RMP *fam-a* family is shown in **Fig 1**, where branches with nodes that are supported by bootstrap values >75 are shown in bold. The topology is relatively well resolved and most terminal nodes are robust. Although basal nodes are less robust, significant bootstrap values were recovered for the major clades (black squares in **Fig 1)**. The gene number differs between the RMP with *P*. *berghei* ANKA having significantly fewer genes (45) than *P*. *chabaudi chabaudi* AS (134) or *P*. *yoelii* YM (113)(**S1 Table**). The distribution of genes from each species in the tree is punctate, i.e. they are not monophyletic but are found throughout the tree indicating the presence of multiple ancestral lineages. These ancestral lineages (a total of 20) are represented by the different clades that contain orthologous genes from the three RMP (orthologous loci are labelled green in **Fig 1**). In seven cases there has been a secondary loss in one species (small black crosses in the green bars in **Fig 1**). The putative orthologs from all clades have a conserved location in the RMP genomes, confirming their relatedness. However, in *P*. *chabaudi* and *P*. *yoelii* most genes are species-specific paralogs (87% in both species), that are more closely related to *fam-a* genes in the same genome than to *fam-a* genes in the genome of another RMP, emphasising that considerable gene duplication occurred after RMP speciation.

**Fig 2:**
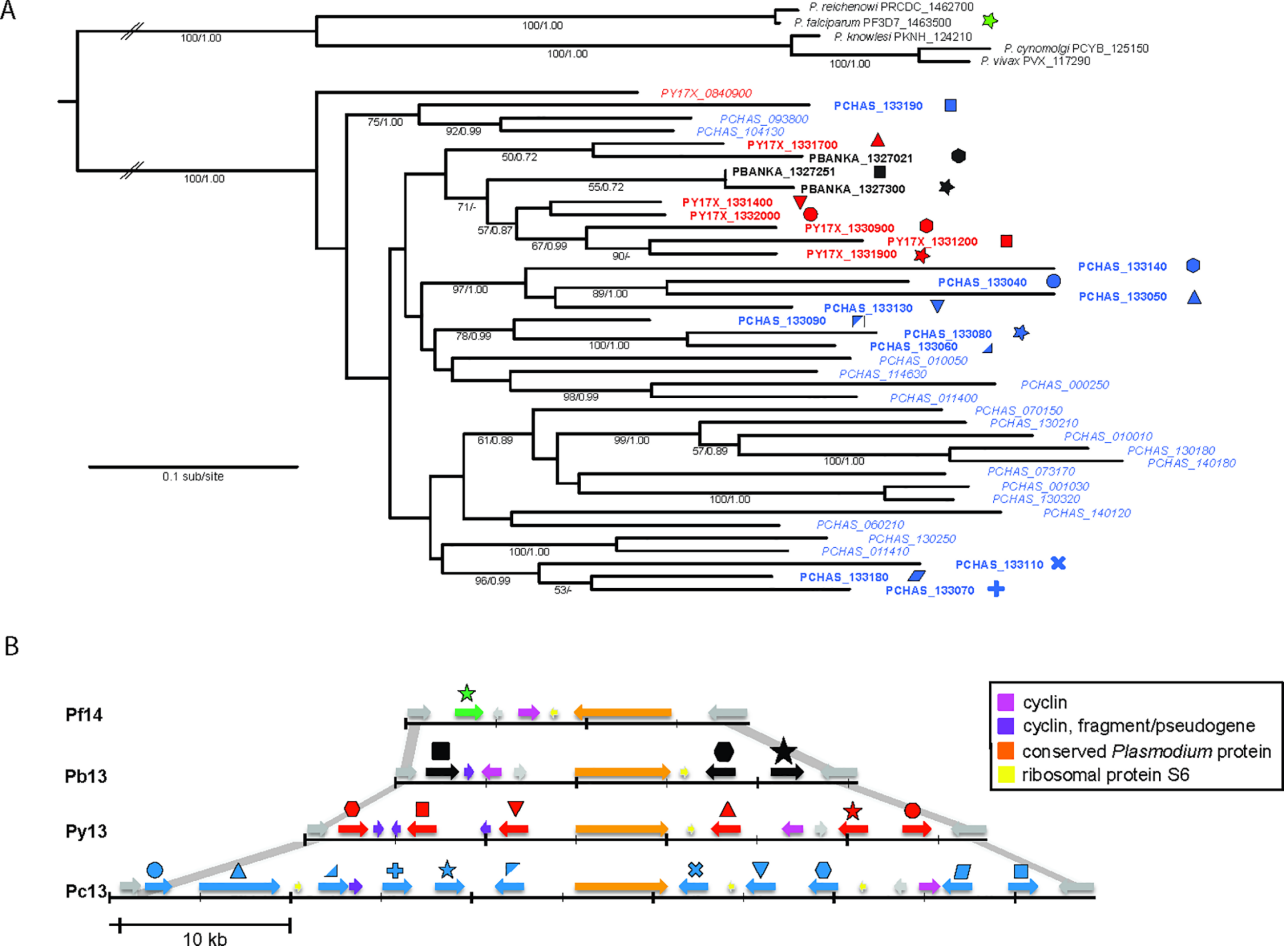
Maximum likelihood phylogeny and chromosomal location of *fam-a* genes that have an internal, syntenic chromosomal location **A.** The tree was estimated using RAxML and a GTR+Γ model. Nodes support is indicated by bootstrap proportions and Bayesian posterior probabilities. Genes that occupy the conserved, chromosomal-internal locus are labelled in bold and accompanied by a symbol, colour-coded by species (repeated in **B**). In *P*. *yoelli* and *P*. *chabaudi*, these genes are paraphyletic with other genes that have been transposed to subtelomeric locations of other chromosomes (these paralogs are labelled in italics). The tree is rooted with the single copy *fam-a* orthologs from primate *Plasmodium* spp.**B.** Chromosomal organisation of the internal copies of *fam-a* genes on chromosome 14 of *P*. *falciparum* 3D7 (Pf14; green), chromosomes 13 of *P*. *berghei* ANKA (Pb13; black), *P*. *yoelii yoelii* 17X (Py13; red) and *P*. *chabaudi* AS (Pc13; blue). *Fam-a* genes are interspersed with cyclin (lilac), cyclin fragments (violet), ribosomal protein S6 (yellow) and conserved *Plasmodium* protein (orange). The internal *fam-a* region is bordered by RNA polymerase III subunit RPC4 (PF3D7_1463400, PBANKA_1327000, PY17X_1330800, PCHAS_133030) and YL1 protein (PF3D7_1464000, PBANKA_1327400, PY17X_1332100, PCHAS_133200) (shown in grey). Synteny between RPC4 and YL1 is shown with grey lines. The arrows indicate the location on forward and reverse strands.

The RMP *fam-a* phylogeny can be robustly rooted since primate malarias contain a single copy *fam-a* gene [33], thereby providing an ideal outgroup. The primate *fam-a* genes share a syntenic, internal chromosomal location and all homologs cluster robustly together in the tree (**Figs 1 and 2A)**. These out-group *fam-a* genes lack a signal peptide, whereas in the RMP *fam-a* genes a predicted signal peptide is linked to the conserved PYST-A domain via a low complexity region of variable length. All three RMP have a cluster of several *fam-a* genes on chromosome 13 that share a syntenic, internal location with the *fam-a* genes of primate malarias (www.GeneDB.org; **Fig 2B**) with which they form a robust clade (**Fig 2)**. Unlike the human primate malaria species, the RMP loci with internal *fam-a* copies show diversity with respect to the location and number of *fam-a* genes (**Fig 2B**), indicating that rearrangements and duplications of *fam-a* genes occurred after speciation in this internal locus. Although the internal RMP *fam-a* genes on chromosome 13 cluster together in the phylogenetic tree, they do not cluster tightly with the outgroup (**Fig 1**). It is therefore unclear from the tree topology which of the (internal) RMP *fam-a* loci is the ancestral gene from which all others were derived.

In conclusion, our phylogenetic analysis shows that evolution of the *fam-a* family in RMP species occurred in two phases. Firstly, gene duplication in the common RMP ancestor has created 20 gene loci that maintain orthology across RMP species and are conserved in structure and genomic location. Secondly, subsequent independent expansions have created abundant species-specific clades, mainly in *P*. *chabaudi* and *P*. *yoelii*. The presence of structurally different and conserved Fam-a proteins may indicate that conserved, functional differences may exist between family members in the three RMP as has been suggested for members of the *pir* family [9, 33, 38].

**Fig 3:**
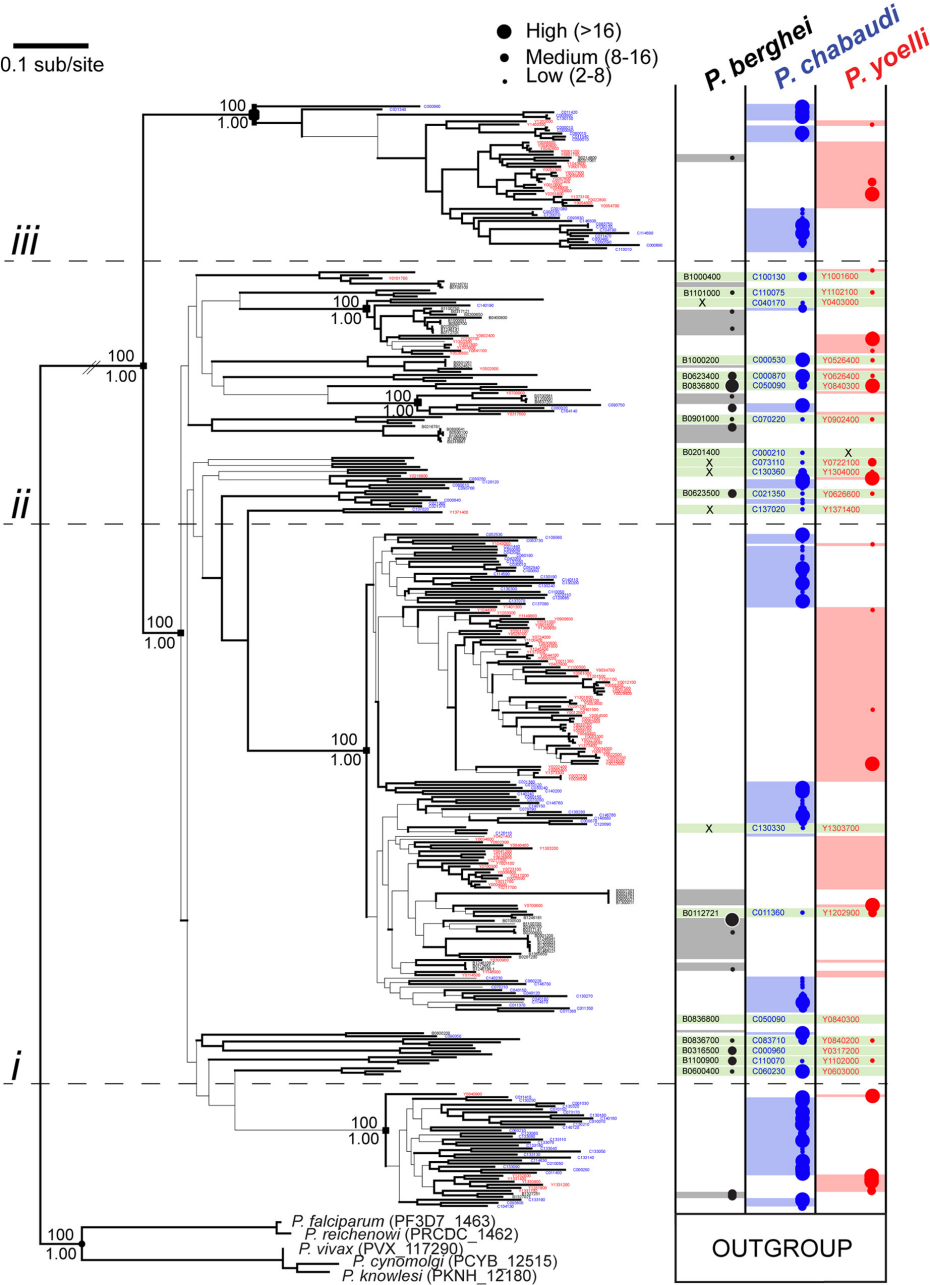
Maximum likelihood phylogeny of *fam-b* gene sequences from *Plasmodium* spp. The tree was estimated using RAxML and a GTR+Γ model. Branches subtended by nodes with >75 bootstrap support are shown in bold. Robust basal nodes are indicated by black squares with bootstrap proportions (above node) and Bayesian posterior probabilities (beneath node). At right, coloured blocks indicate the species to which a terminal node belongs. Clades of orthologs that display positional conservation are indicated with green blocks; where a sequence has been lost secondarily in one species, this is shown by an ‘X’. The phylogeny is subdivided into four sections: divergent genes included conserved loci, placed at the root of the tree (below line *i*); predominantly *P*. *yoelli* species-specific genes *P*. *berghei*- and *P*. *yoelli*-specific paralogs (between lines *i*, *ii* and *iii*); and predominantly *P*. *chabaudi* species-specific genes (above line *iii*). Transcription levels (shown as different coloured and sized circles) in blood stages are shown for individual genes based on RNAseq data (FPKM values) (from [33] and **S1** Table). Expression levels as shown by four different sized circles: Class 1 (smallest circle): 2-8x the threshold level; class 2: 8-16x the threshold; class 3 (largest circle): >16x the threshold.

In contrast to the phylogenetic trees of *fam-a* and *pir* families, the *fam-b* tree contains only a few branches with nodes that are supported by bootstrap values >75 (even at some terminal nodes; **Fig 3**). The *P*. *berghei* ANKA *fam-b* family consist of 48 members, whereas *P*. *c*. *chabaudi* AS and *P*. *yoelii* YM have 26 and 53 members, respectively (**S1 Table**). All *fam-b* genes are located in the subtelomeric regions and comprise two exons and one intron, encoding proteins of approximately 260 amino acids. They are characterized by the presence of the PYST-B domain and most members have a predicted signal peptide, PEXEL motif and transmembrane domain (www.GeneDB.org; [33]. The majority of *fam-b* genes (above line *i* in **Fig 3**) are separated from a small group of atypical sequences that have long branches. Most of these atypical sequences are found at four loci conserved in all three species. Orthology is very rare in the other *fam-b* genes. Instead, there are multiple, species-specific clades that are paraphyletic with heterospecific genes, suggesting that ancestral lineages have diversified independently after RMP speciation. In short, the phylogeny indicates that each RMP has expanded its *fam-b* repertoire independently through duplication of only a few (at least two) ancestral lineages. In contrast to the *fam-a* family, there are no *fam-b* homologs in non-RMP that could be used as an outgroup. Therefore, it cannot be stated unambiguously that the atypical *fam-b* genes represent the ancestral RMP genes. However, their positional conservation in RMP and the distinct structure may indicate that these represent ancestral lineages that have not been modified subsequently.

### Transcription of fam-a, fam-b and pir genes in blood-stage populations

**Fig 4:**
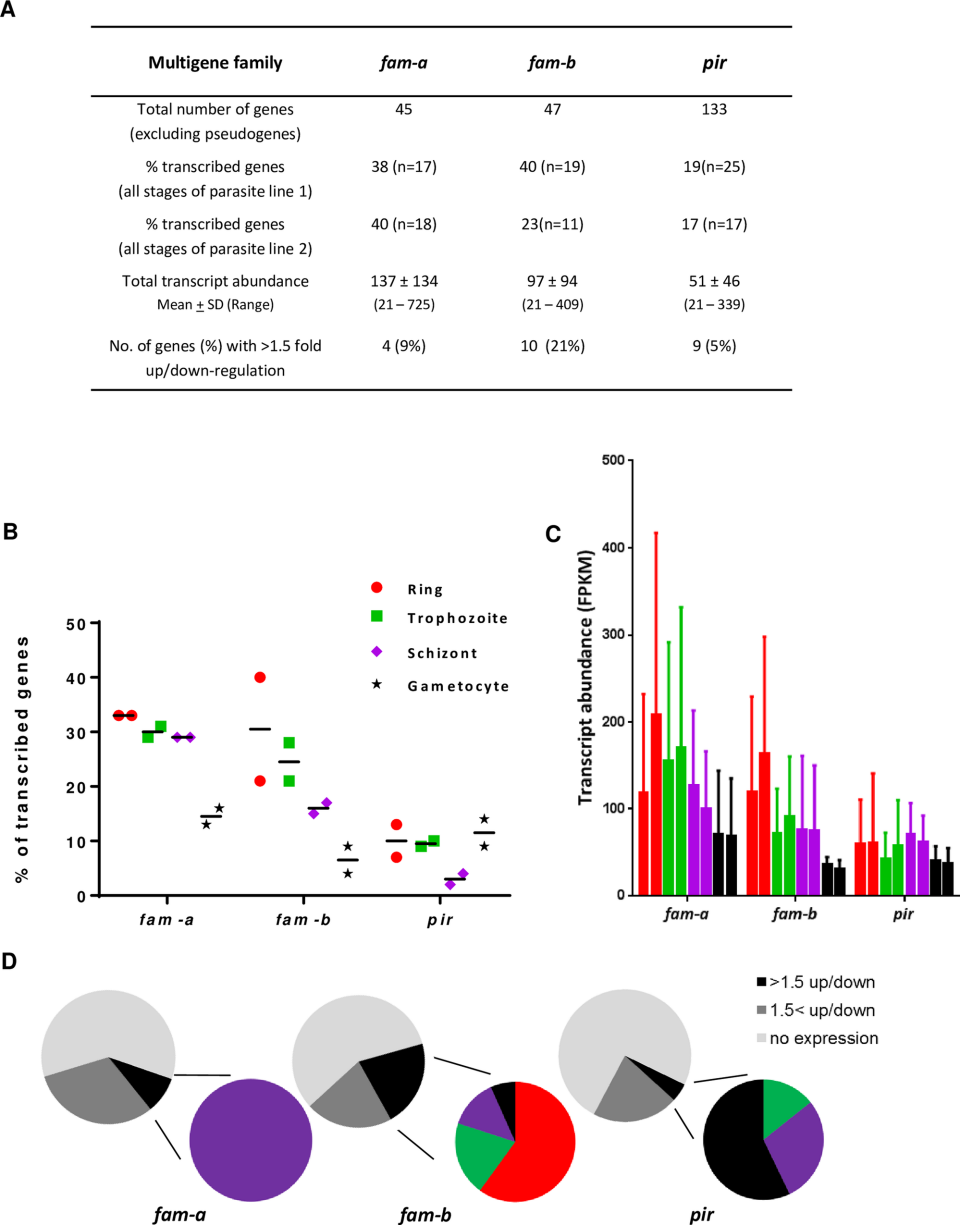
Transcription of *pir*, *fam-a* and *fam-b* genes based on RNAseq data of different blood stages of two *P*. *berghei* ANKA reference lines. **A**. Features of transcription of *pir*, *fam-a* and *fam-b* genes in the two *P*. *berghei* reference lines (line 1 and line 2) based on RNAseq data (from[33] and shown in S1 Table). Transcribed genes are genes with an FPKM value above the cut-off level of 21. Total transcript abundance is the sum of all FPKM values observed in the different blood stages (see **B**). The fold up-down regulation is based on the difference in FPKM values of individual genes between blood stages of the two different parasite lines (see **S1 Fig**). **B.** Percentage of genes transcribed in the different blood stages (see **A**). Ring, red; trophozoite, green; schizont, purple; gametocyte, black. **C.** Total transcript abundance in the different blood stages: mean and standard deviation of total transcript abundance of all FPKM values observed in the different blood stages (see **A**). **D.** Percentage of non-transcribed genes (light grey) and genes with less (grey) or more (black) than 1.5x difference in transcript abundance between blood stages of two different parasite lines (see **A**). The coloured circles show the genes with >1.5 fold down-or upregulation in the four different blood stages (see **B**).

To obtain more insight into the transcriptional activity of members of the RMP multigene families we analysed existing genome-wide RNAseq data of blood-stage parasites [33]. For the *P*. *berghei fam-a*, *fam-b* and *pir* families we first re-analysed the RNAseq data using the improved annotation described above (see **S1 Table** for RNAseq FPKM values of all genes). Visualization of the transcript levels of all *fam-a* and *fam-b* genes in the phylogenetic trees shows that multiple members of nearly all phylogenetic clades are transcribed in RMP blood-stage populations (**Figs 1 and 3**). A similar widespread transcriptional activity of multiple members of different clades in blood stages had also been demonstrated for the *pir* family [33]. For the *pir* family it is known that significant changes in transcriptional activity of individual members can occur during blood-stage infections [8, 33, 42–44] and that mosquito transmission results in large scale changes of transcription levels of many members [45]. Less is known about variation in transcription levels of the *fam-a* and *fam-b* members. We therefore analysed existing RNAseq data of blood-stage populations of two isogenic lines of *P*. *berghei* ANKA [33]. In these populations 23 to 40% of the *fam-a* and *fam-b* members are transcribed (**Fig 4A and 4B**). In both lines the percentage of transcribed *pir* genes is lower, between 17 and 19%. Although the total number of *pir* genes transcribed in blood stages of the two lines is higher than the number of *fam-a* and *fam-b*, the total transcript abundance for the *pir* genes is lower (**Fig 4C**). In gametocytes the percentages of transcribed genes of both the *fam-a* and *fam-b* family are lower in comparison to asexual blood-stages. Remarkably, the percentage of transcribed *pir* genes is higher in the (non-sequestering) gametocytes compared to that of asexual blood-stages (**Fig 4B**).

Both the proportion of transcribed genes and transcript abundance of the families are very similar in in the two *P*. *berghei* ANKA lines (**Fig 4A and 4B**). Since these lines have a different history of blood-stage propagation in the laboratory, these observations suggests the absence of large scale changes in expression of family members during blood-stage propagation. However, when we analyse transcript abundance of individual genes in the two populations, significant differences in transcription of particular genes are observed between these populations (**Fig 4D; S1 Fig**). Five to nine percent of the *pir* and *fam-a* members show a more than 1.5x difference in transcript abundance whereas 21% of the *fam-b* members show a difference in transcript abundance higher than 1.5x (**Fig 4D**).

Combined, our analyses of RNAseq transcription data demonstrate transcriptional activity of a relatively high percentage of *fam-a* and *fam-b* members (from different phylogenetic clades) in populations of blood-stage parasites. In addition, transcript abundance of individual genes of the three families can differ significantly between blood-stage populations of two isogenic lines, notwithstanding the relatively stable proportions of the total number of transcribed genes and the total transcript abundance.

### Protein expression and cellular location of pir, fam-a and fam-b proteins in blood stages

To further analyse expression at the protein level we generated and analysed a number of transgenic parasites expressing a fluorescently-tagged family member. An important criterion for selection of genes for fluorescent tagging was the availability of existing data for transcription and/or protein expression in *P*. *berghei* blood-stage parasites. In **Table 1** the 12 selected members for tagging are shown. For all selected genes, except for one (*pir1*), transcriptome evidence for expression has been reported [33]. In addition, proteome evidence for expression has been reported for all selected *fam-a* and *fam-b* proteins and for three out of eight selected *pirs* (**Table 1;** [36]).

**Table 1:** Features of tagged members of the *pir*, *fam-a* and *fam-b* multigene families

					BLOOD	
Gene ID *P*. *berghei*	Gene product (+ domain)^1^	name	phylogeny(clade)^2^	SP/Pexel/TM^3^	Expression RNAseq (max. FPKM)^4^	Expression Proteome(max. spectra)^5^
PBANKA_0836800	Fam-a (*pyst-a*)	Fam-a1	N.A.	+ / - / -	510 (tz)	>10
PBANKA_1327300	Fam-a (*pyst-a*)	Fam-a2	N.A.	+ / - / -	605 (tz)	>10
						
PBANKA_0316700	Fam-b (*pyst*-b)	Fam-b1	N.A.	+ / + / +	67 (tz)	1
PBANKA_0722600	Fam-b (*pyst*-b)	Fam-b2	N.A.	+ / + / +	390 (r)	1
						
PBANKA_1400300	PIR (*pir*)	PIR1	S4	- / - / +	<21	4
PBANKA_0837161	PIR (*pir*)	PIR2	S4	- / - / +	49 (gct)	N.D.
PBANKA_0500200	PIR (*pir*)	PIR3	S8	- / - / +	487 (r)	>10
PBANKA_1200400	PIR (*pir*)	PIR4	S1	- / - / +	55 (g)	N.D.
PBANKA_1300200	PIR (*pir*)	PIR5	S6	- / - / +	310 (r)	5
PBANKA_0216000	PIR (*pir*)	PIR6	L2	- / - / +	37 (sz)	N.D.
PBANKA_1040561	PIR (*pir*)	PIR7	L2	- / - / +	129 (r)	N.D.
PBANKA_0524600	PIR (pir)	PIR8	N.A.	- / - / +	116 (tz)	N.D.

SP: signal peptide; TM: transmembrane domain; tz: trophozoite; r: ring form; gct: gametocyte; sz: schizont; N.A.: not applicable; N.D.: not done

^1^ www.GeneDB.org

^2^ from: Otto et al, 2014

^3^ from: Pasini et al., 2013 and www.PlasmoDB.org

^4^ FPKM values in *P*. *berghei* blood stages (from: Otto et al, 2014)

^5^ no. of spectra in proteome analyses of *P*. *berghei* blood stages (from Pasini et al., 2013)

Transgenic parasites containing a single fluorescently-tagged member were generated by standard genetic modification technologies for *P*. *berghei* [46]. Parasites were transfected with linear constructs that introduce the fluorescent tag at the C-terminus of the endogenous gene by integration through single cross-over homologous recombination. The generation of eight of the twelve single-gene tagging (SGT) mutants have been reported previously by Pasini *et al*. (**S2 Table**; [36]). In this study we tagged four additional *pir* genes. For two genes that were tagged before with mCherry, we generated additional mutants that have GFP-tagged versions of the same gene (**Table 2**). In addition to tagging of multigene family members we tagged two blood-stage exported proteins encoded by single copy genes, i.e. *smac* [16] and *ibis1* [47] (**Table 2**). The tagging of *ibis1* was performed as described for the other SGT mutants. Details of the generation and genotyping of all mutants have been published in the RMgmDB database (www.pberghei.eu; **S2 Table**).

**Table 2:** Expression of proteins encoding blood-stage exported proteins in blood- and liver-stages

				BLOOD				LIVER
Name tagged protein	Fluorescent tag	Mutant name	RMgmDB ID^1^	expression andlocalisation	% of clones fluorescent	fluorescent before passage (%)	fluorescent after passage (%)	proteinlocalisation
**Single gene-tagging mutants (multigene families)**				** **	** **	** **	** **	
Fam-a1	mCherry	1477cl3	690	Yes; RBC s	33% (n = 3)	80–90%	50–70%	Yes; PV
Fam-a1	GFP	1941	1283	Yes; RBC s	N.A.	25–30%	30%	No
Fam-a2	mCherry	1448cl2	693	Yes; RBC c pa	60% (n = 5)	99%	85–100%	Yes; PV
Fam-b1	mCherry	1599cl4	699	Yes; RBC c pa	66% (n = 3)	5–15%	5%	Yes; PV
Fam-b2	mCherry	1731cl4	700	Yes; RBC c pa	100% (n = 5)	65%	50–70%	Yes; PV
Fam-b2	GFP	1942	1282	Yes; RBC c pa	N.A.	50–75%	N.D.	N.D
PIR1	mCherry	1531cl3	695	Yes; RBC c pa	100% (n = 5)	15%	60–70%	Yes; PV
PIR1	mCherry	1944cl1	1281	Yes; RBC c pa	66% (n = 3)	10–20%	N.D.	N.D.
PIR2	GFP	603cl3	696	Yes; RBC c pa	75% (n = 4)	5–10%	5%	No
PIR3	mCherry	1918cl4	697	Yes; RBC c pa	25% (n = 4)	1%-5%	N.D.	No
PIR4	mCherry	2450	1233	Yes; RBC c pa	N.A.	0.1–2%	N.D.	No
PIR5	mCherry	2448cl1	1234	Yes; RBC c pa	100% (n = 3)	25–50%	N.D.	N.D.
PIR6	mCherry	1892	698	Yes; RBC c pa	N.A.	<0.1%	N.D.	N.D.
PIR7	mCherry	2211	1235	Yes; RBC c pa	N.A.	<0.1%	N.D.	N.D.
PIR8	mCherry	2312, 2313	1236	Yes; RBC c pa	N.A.	50–60%	30–60%	yes, parasite cyt
**Double gene-tagging mutants (multigene families)**								
Fam-a2 Fam-a1 *Fam-a2/a1*	mCherryGFPmCherry&GFP	2010 (2011)	1244	Yes; RBC c paYes; RBC s	N.A.	70–80%40–65%40–55%	70–80%45–50%30–40%	Yes; PV (>90%)No
Fam-a2 Fam-a1 *Fam-a2/a1*	GFPmCherryGFP&mCherry	2504cl3	1245	Yes; RBC c paYes; RBC s	100% (n = 3)	80–90%80–90%80–85%	75–80%60–65%70–80%	Yes; PV (70–75%)Yes, PV (30–40%)30–40%
Fam-b1 Fam-b2 *Fam-b1/b2*	mCherryGFPmCherry&GFP	2421(-2424)	1246	Yes; RBC c paYes; RBC c pa	N.A.	40–50%40–45%35–45%	30–50%50–80%20–40%	Yes; PVNo
PIR1 PIR3*PIR1/PIR3*	mCherryGFPmCherry&GFP	2020 (2021)	1247	Yes; RBC c paYes; RBC c pa	N.A.	35–45%25–35%20–30%	40–60%1–20%1–5%	Yes; PVNo
**Single gene-tagging mutants (single copy genes)**								
IBIS1	GFPmcherry	20091940cl1	1237	Yes; RBC c pu	N.A.100% (n = 1)	N.D>90%	N.D.N.D.	Yes; PV (>90%)
SMAC	mCherry	1565cl1	1238	Yes; RBC c pa	100% (n = 4)	>90%	N.D.	Yes; PV (>90%)

^1^ www.pberghei.eu

N.A.: not applicable; N.D.: not done; RBC: red blood cell; s: surface; cytoplasm patchy: c pa; cytoplasm punctuate: c pu; PV: parasitophorous vacuole

**Fig 5:**
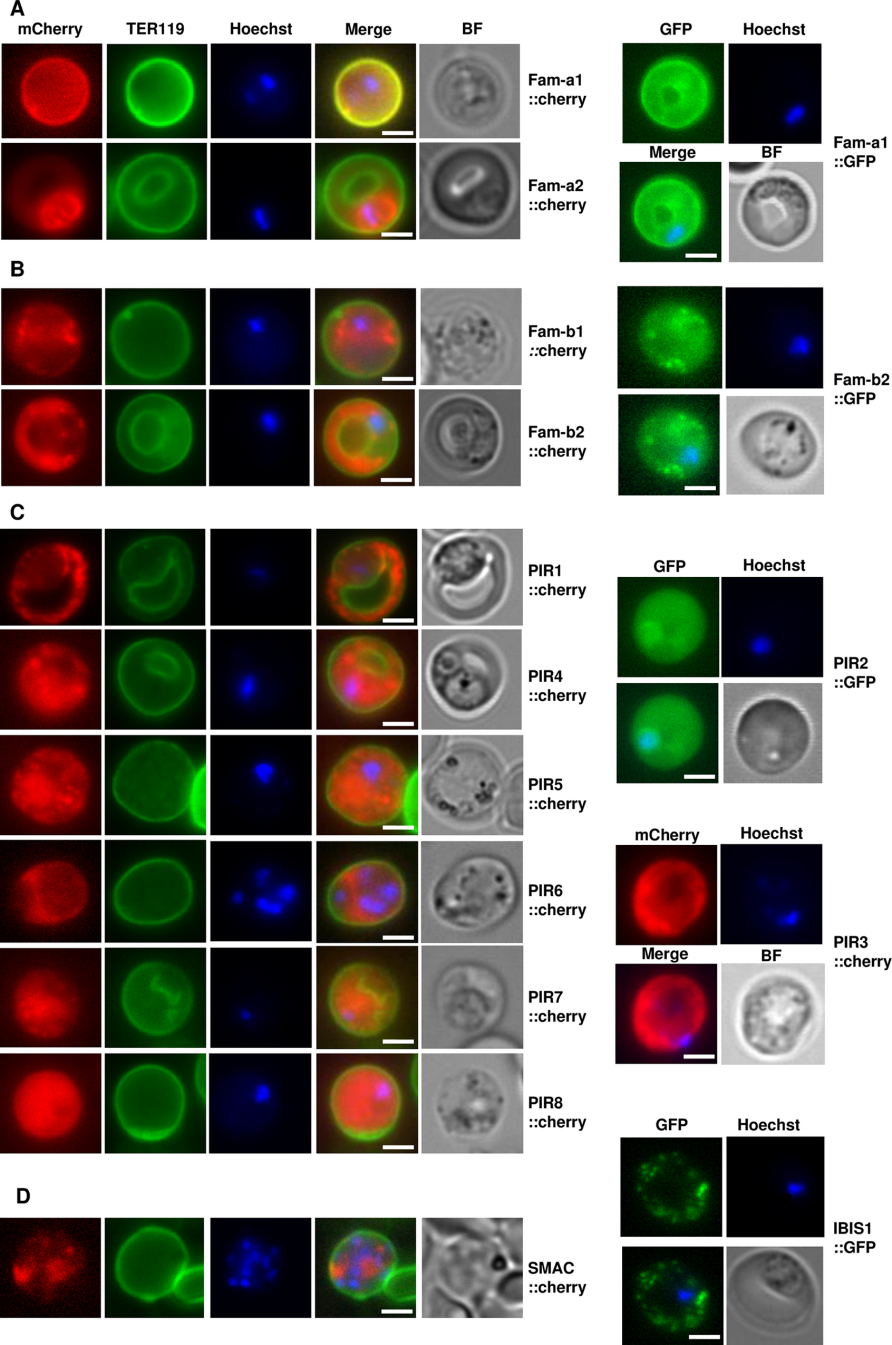
Expression and export of fluorescently-tagged *fam-a* (**A**) *fam-b* (**B**) and *pir* (**C**) members in blood stages of single-gene tagging (SGT) mutants. In **D** expression and export is shown of the fluorescently-tagged proteins SMAC and IBIS that are encoded by single copy genes. The plasma membrane of the red blood cell is stained with TER119 antibodies (green) and parasite nuclei are stained with Hoechst. BF: bright field. Scale bar: 2μm.

All SGT mutants showed expression of the fluorescently-tagged protein in blood-stage parasites and these proteins were exported to the RBC cytoplasm (**Table 2; Fig 5**). For 8 of the 12 proteins export in blood-stages had been shown before. Fluorescence microscopy analysis of live cells revealed that most of these proteins have a punctate/patchy or diffuse localisation pattern within the iRBC cytoplasm. For only one *fam-a* member (Fam-a1, EMAP1) we observed a fluorescence-staining pattern indicative for a location at the iRBC surface membrane ([36]; **Fig 5**). The additional four *pir* genes (PIR4, 5, 7 and 8) that were tagged in this study were also exported into the RBC cytoplasm and showed a patchy or diffuse localisation pattern with the iRBC cytoplasm (**Fig 5**) as had been observed for all other tagged *pirs*. The single-copy genes *smac* and *ibis* that were tagged were also exported into the RBC cytoplasm with a pathchy/punctate localisation pattern (**Figs 5D and S2**).In the uncloned blood-stage populations of most mutants more than 5% of the parasites were fluorescent-positive (**Table 2).** Only in two mutants (with tagged versions of *pir6* and *pir7)* the percentage of fluorescent blood-stages was very low (less than 0.1%). Both *pirs* belong to the ‘large-form’ *pirs* [33].

To further analyse expression we first cloned all SGT mutants, except for three mutants that had a low percentage of fluorescent parasites in the parent population (*pir4*, *pir6* and *pir7*). For all cloned SGT mutants, we were able to obtain one or more clones that produced fluorescent-positive blood-stages. However, for multiple SGT mutants we also selected clones that did not show any fluorescent blood-stages (**Table 2**), despite having a tagged gene as shown by genotyping through Southern analysis of separated chromosomes (**S3 Fig**). The selection of fluorescent-negative clones suggests a relatively high rate of ‘expression switching’ of these family members resulting in silencing of expression.

We next determined the percentage of fluorescent blood-stages of the fluorescent-positive clones after a period of 1–2 weeks of asexual multiplication (7–14 asexual cycles) and in blood-stages after passage through mosquitoes. In the blood-stage populations before mosquito passage the percentage of fluorescent parasites ranged between 5 and 99% (**Table 2).** The presence of significant numbers of non-fluorescent parasites in the clonal populations is in line with a relatively high switching rate of expression and is in agreement with significant differences in transcript levels of individual genes between the blood-stage populations of two isogenic *P*. *berghei* ANKA lines (**S1 Fig**).

Eight of the nine cloned SGT mutants were passaged through mosquitoes. After mosquito passage we observed fluorescent-positive parasites in blood-stage populations of all 8 mutants. The percentage of fluorescent blood-stages after mosquito passage was in most mutants comparable to that before passage (**Table 2**). Only for the mutant expressing tagged PIR1 we found a strong increase of the percentage of fluorescent parasites after mosquito passage (an increase from 15 to 60–70%; **Table 2**). These observations indicate that mosquito passage does not result in resetting of expression that would result in large scale differences in expression levels between blood-stages before and after mosquito passage.

To analyse whether significant changes in expression of individual members occur during prolonged blood-stage infections *in vivo*, we determined the percentage of fluorescent parasites during infections in Brown Norway rats of three cloned mutants (expressing a tagged *fam-a*, *fam-b* and *pir* gene). *P*. *berghei* infections in Brown Norway rats show a characteristic course of parasitemia with one or two early peaks with a maximal parasitemia of less than 5%, after which the rats are able to control the infections with occasional small waves of recrudescent parasitemias, ranging between 0.01–0.5% (**S4 Fig**). During these prolonged infection periods (38–51 asexual multiplication cycles) the *fam-a* and *fam-b* mutants showed relatively high and stable percentages of fluorescent parasites. Only in the *pir1* mutant did this percentage dropped significantly in both rats (from 30% to lower than 10%; Two-way ANOVA; p = 0.0062;**S4 Fig**).

The observations of relatively stable percentages of fluorescent parasites before and after mosquito transmission and during prolonged periods of asexual multiplication demonstrate the absence of large changes in expression levels of these members at a population level. However, the observed differences in transcript levels of individual genes in blood-stage populations of two isogenic *P*. *berghei* ANKA lines (**S1 Fig**) and the selection of fluorescent-negative clones of SGT mutants (**Table 2**) demonstrate that significant changes can occur in expression levels of individual genes.

### Simultaneous expression in a single blood-stage parasite of two fluorescently-tagged proteins of the same family

The expression of most of the tagged proteins in a relatively high percentage of blood-stage parasites suggests that a single parasite may express simultaneously multiple members of the same family. To obtain formal proof for simultaneous expression of multiple members in a single blood-stage parasite, we generated four ‘double gene tagging’ mutants (DGT) that contain two fluorescently-tagged genes from the same family (**Table 2**). These DGT mutants were generated by transfection of SGT mutants with linear constructs for C-terminal tagging by single cross-over homologous recombination. These constructs contain the *hdhfr* selectable marker that allows for selection of the DGT with the drug WR99210 [48]. The four DGT mutants contain the following pairs of genes tagged with either mCherry or GFP: *fam-a1/fam-a2* (2 independent mutants; RMgm ID 1244 and 1245), *fam-b1/fam-b2* (RMgm ID 1246) and *pir1/pir3* (RMgm ID 1247; **Table 2**).

**Fig 6:**
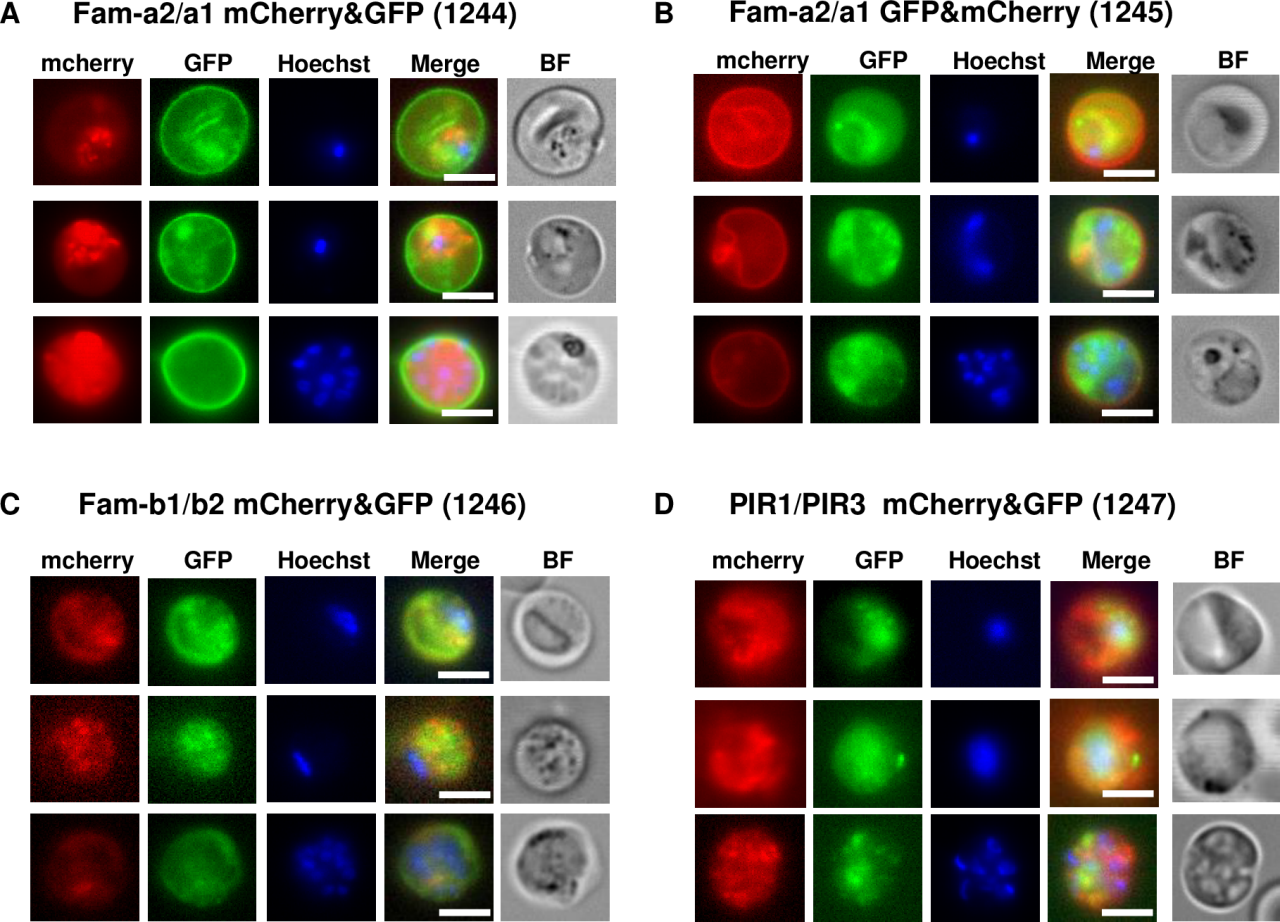
Simultaneous expression (and export) of two proteins of the same family in a single blood stage parasite (trophozoites or schizonts) of double-gene tagging (DGT) mutants. These mutants contain the following pairs of genes tagged with either mCherry or GFP: *fam-a1/fam-a2* (2 independent mutants; panel A, B), *fam-b1/fam-b2* (panel C*) and pir1/pir3* (panel D); RMgm ID as indicated in **Table 2**. Localisation of mCherry-tagged members in the trophozoite stage (upper two rows) and in the schizont (low row) stage. Parasite nuclei are stained with Hoechst, BF bright field. scale bar: 5μm.

In all mutants we detected blood-stage parasites (trophozoites, schizonts) that expressed and exported the two tagged proteins simultaneously (**Fig 6**). For the *fam-a* and *fam-b* DGT mutants, 20–90% of the parasites expressed both proteins simultaneously. For the *pir* DGT mutant this percentage ranged between 1 and 3%. The two tagged *fam-a* members had a different cellular location in a single parasite. In both independent *fam-a1/fam-a2* DGT mutants, Fam-a1 is located at the RBC surface membrane whereas Fam-a2 locates in the RBC cytoplasm. The same difference in localisation we had also observed in blood-stages of the SGT mutants that expressed either Fam-a1 or Fam-a2 (**Fig 5**). The simultaneous expression of multiple family members in a single parasite demonstrates that expression of members of these families is not mutually exclusive as has been shown for the *P*. *falciparum var* gene family [49–51].

### Members of the three multigene families are expressed in liver-stages and are exported into the parasitophorous vacuole

We next analysed the expression of several tagged proteins throughout development in the mosquito and during development in the liver. Parasites of both SGT and DGT mutants were fed to *Anopheles stephensi* mosquitoes and oocysts (day 8 to 12) and salivary gland sporozoites were analysed for expression of the tagged proteins by fluorescence microscopy. In all mutants analysed, six SGT (*Fam-a1*, *Fam-a2*, *Fam-b1*, *Fam-b2*, *PIR1*, *PIR8*) and the four DGT mutants, we did not observe any fluorescence-positive oocysts or sporozoites. After mosquito passage, all SGT and DGT mutants expressed the tagged proteins in blood-stage populations (**Table 2**) demonstrating that the absence of expression in oocysts and sporozoites is only a temporarily switch-off of expression during development in the mosquito.

**Fig 7:**
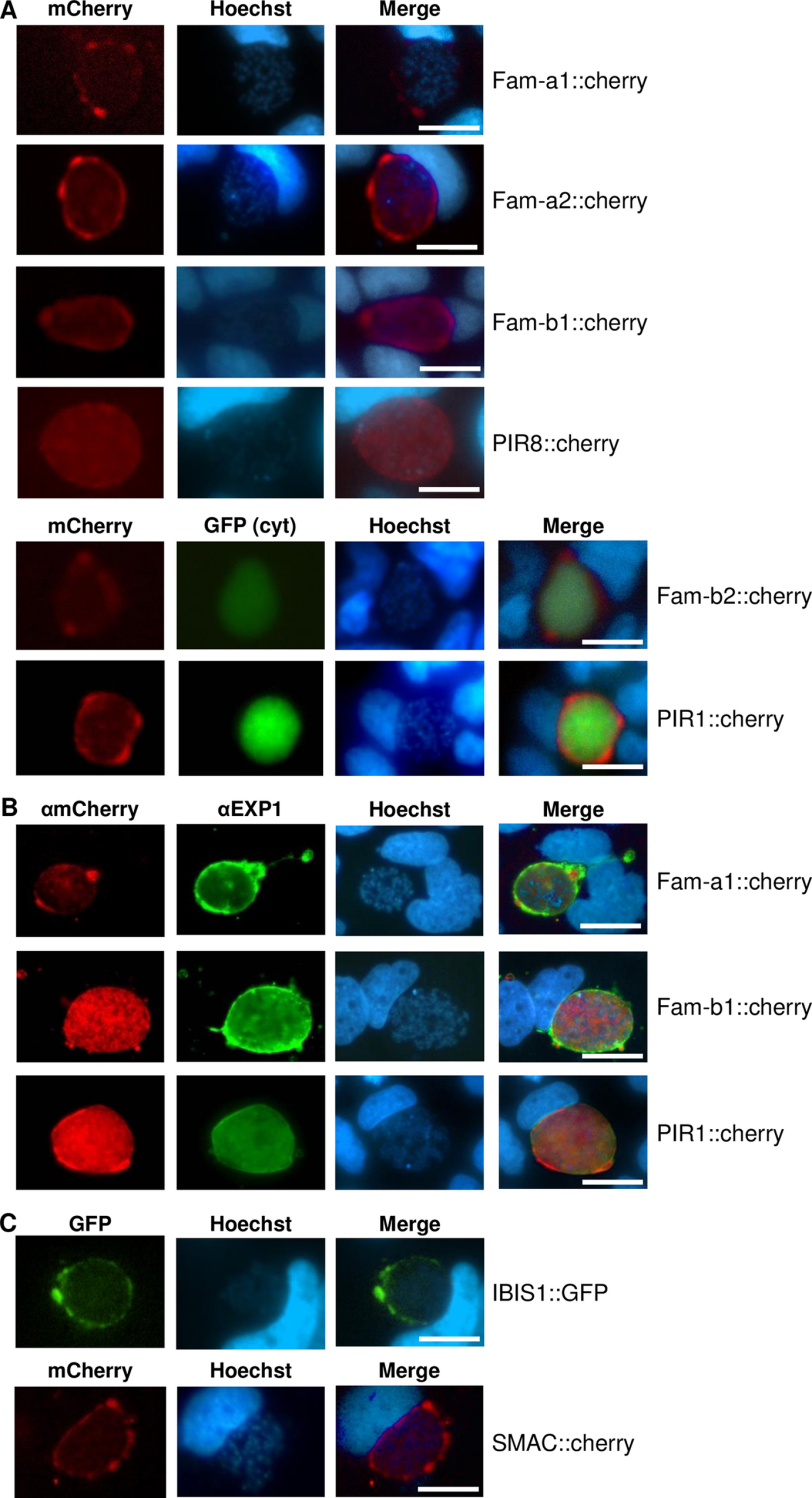
Expression of fluorescently-tagged proteins of multigene families in liver stages of single-gene tagging (SGT) mutants at 48hpi in cultured hepatocytes (Huh7). **A**. Fluorescence-microscopy analysis of members of the *fam-a*, *fam-b* and *pir* multigene family in live liver-stages. The parasites expressing mCherry-tagged Fam-b2 and PIR1 also express cytoplasmic GFP (cyt GFP; green). **B**. IFA-analysis of fixed liver-stages using anti-mCherry (red) anti-PbEXP1 (green) antibodies. PbEXP1 is a parasitophorous vacuole membrane resident protein. **C** Fluorescence-microscopy analysis of expression of SMAC and IBIS, exported proteins encoded by single-copy genes in live liver-stages. Nuclei are stained with Hoechst-33342 (blue); scale bar: 10μm.

**Fig 8:**
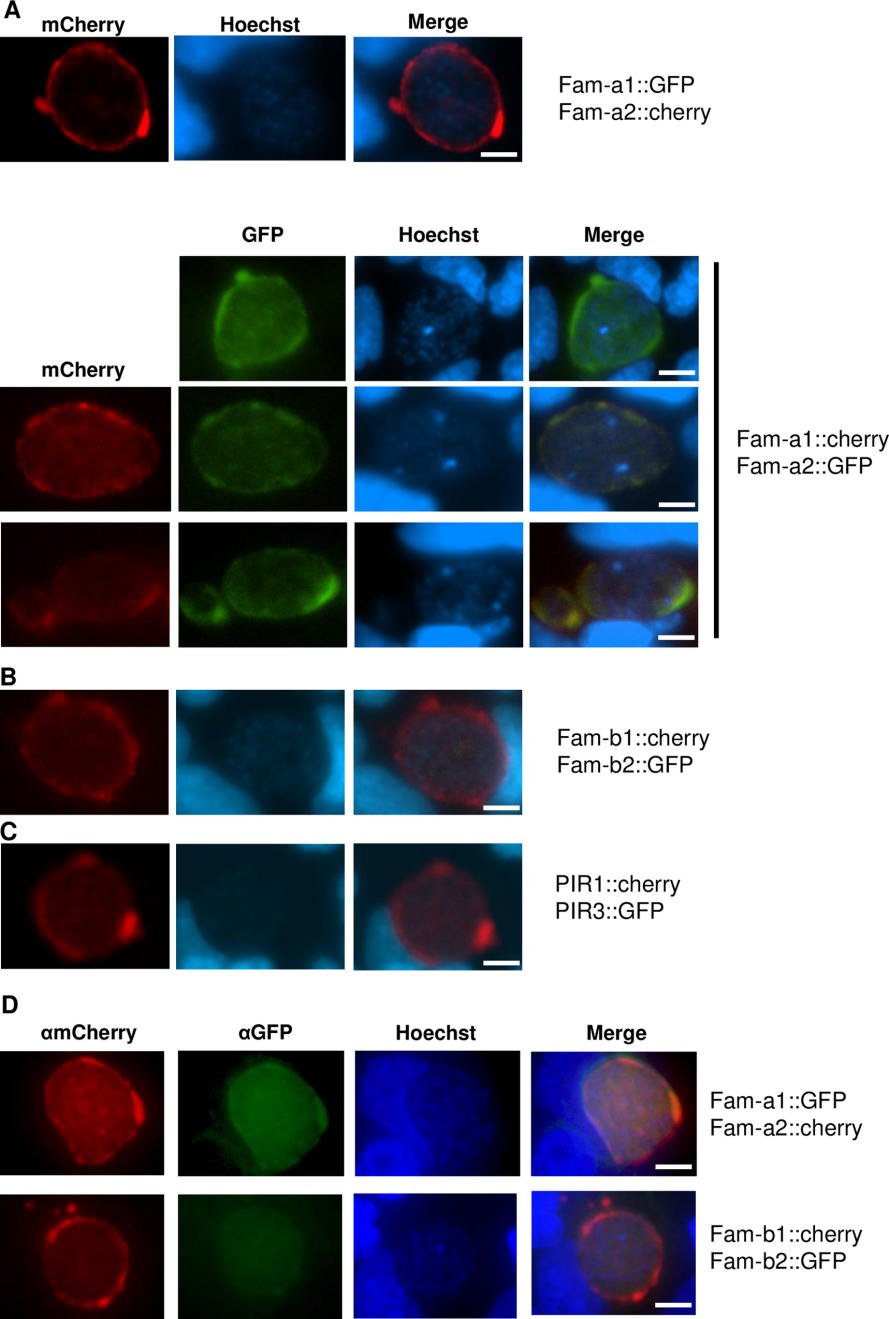
Expression of fluorescently-tagged proteins in liver stages of double-gene tagging (DGT) mutants at 48hpi in cultured hepatocytes (Huh7). Fluorescence-microscopy analysis of members of the *fam-a* (**A**), *fam-b* (**B**) and *pir* (**C**) multigene family in live liver-stages of DGT mutants; we were only able to detect both fluorescently-tagged proteins in only one DGT mutant, fam-a1cherry/fam-a2GFP (in 40–45% of the parasites). **D**. IFA analysis of fixed liver-stages of DGT mutants using anti-GFP (green) and anti-mCherry (red) antibodies. Nuclei are stained with Hoechst-33342 (blue). Scale bar: 10μm.

During the first 30 hours of liver-stage development in cultured Huh7 cells no fluorescence could be detected in all SGT and DGT mutants. However, between 40 and 48 hour after infection of hepatocytes, we observed expression of Fam-a, Fam-b and PIR proteins (**Fig 7**). In five out of six SGT mutants we observed expression in maturing liver-schizonts (**Table 2**, **Fig 7**). Also liver-stages of the DGT mutants were fluorescent-positive (**Fig 8A–8C**) and for one DGT mutant, *fam-a1/fam-a2*, we observed simultaneous expression of both members in 40–45% of the liver-schizonts by live imaging of infected hepatocytes (**Fig 8A**). In addition, we detected simultaneous expression of the two members in the *fam-b1/fam-b2* DGT by immunofluorescence analysis of fixed liver-stages using anti-GFP and anti-mCherry antibodies (**Fig 8D; Table 2**). As observed for the blood stage, multiple family members are simultaneously expressed in a single liver-stage parasite, indicating that also at this stage of development there is an absence of mutually exclusive expression of members of these families. In the *pir1/pir3* DGT we only observed expression of PIR1::mCherry, both by live imaging of infected hepatocytes and by immunofluorescence analysis of fixed parasites using both antibodies.

All tagged proteins were exported into the parasitophorous vacuole (PV), except for PIR8 which was located in the cytoplasm of the parasite. We did not detect fluorescence signals in the cytoplasm of the host cell, both by live imaging and by staining of fixed cells with anti-mCherry or GFP antibodies. The single-copy genes *smac* and *ibis* were also expressed in liver-stages and both proteins were located in the PV. For IBIS a PV location in infected hepatocytes had been shown before [47].

With standard fluorescence microscopy it is difficult to determine whether the proteins are located in the lumen of the PV or are located on/at the PV membrane (PVM). For two Fam-a members we analysed the location in more detail using confocal microscopy. In these analyses we stained the parasites with anti-mCherry recognizing the tagged fam-a proteins and with antibodies against two known PVM-resident proteins (EXP1 and UIS4). A clear overlap was observed between the staining of the PVM-resident proteins and the anti-mCherry staining location indicating that these members are indeed located on/at the PVM (**S5 Fig**). Follow up studies are required to more clearly define the location of the different family members in the PV/PVM and also to explore the possibility that members of the same family may be present either at the PV or the PVM, much like fam-a members are able to locate to different locations in the iRBC (i.e. RBC cytoplasm or RBC plasma membrane).

### A subset of Fam-A proteins show phosphatidylcholine transfer activity *in vitro*

**Fig 9:**
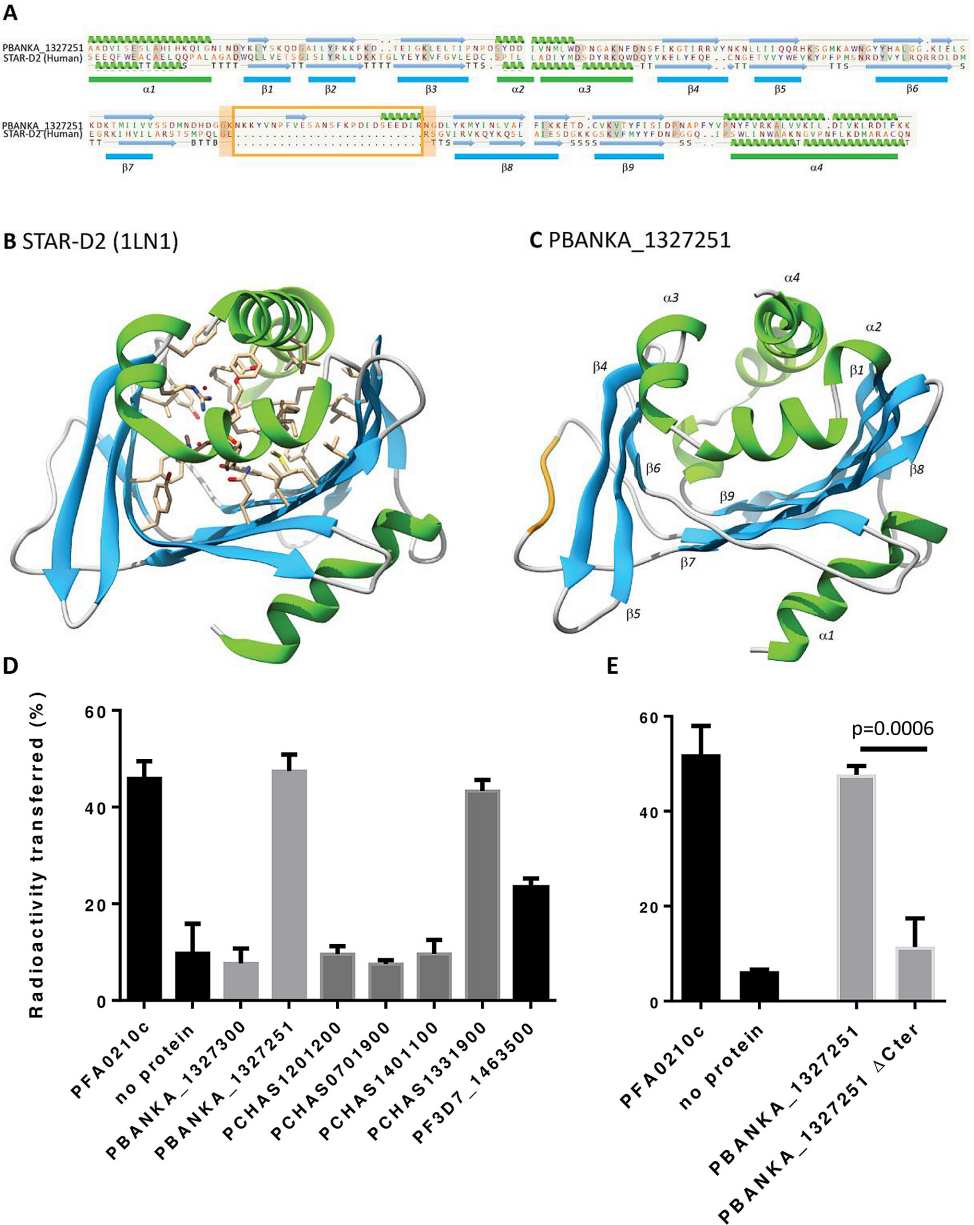
Predicted structure of the START domain of fam-a2 and Phosphatidylcholine (PC) transfer activity of selected recombinant Fam-a proteins. **A**. Secondary structure of Fam-a protein PBANKA_1327251 threaded against the resolved structure of the STAR-D2 domain in the human phosphatidylcholine transfer protein (1LN1). The orange box identifies an expanded protein loop between β-sheets 7 and 8 in Fam-a. **B**. Predicted tertiary structure of 1LN1 showing the binding pocket of the STAR-D2 domain in complex with the ligand, dilinoleoyl-phosphatidylcholine (shown as stick model). **C**. Predicted tertiary structure of PBANKA_1327251 after computational threading against 1LN1. The orange part shows the position of the expanded protein loop in Fam- a as presented in **A**. Alpha helix (green) and beta pleated sheets (blue) are labelled as described in model 1LN1. **D**. Phosphatidylcholine (PC) transfer activity of selected recombinant proteins of the *P*. *chabaudi* (four proteins) and *P*. *berghei* (2 proteins) Fam-A families and of the single *P*. *falciparum* Fam-a protein (PF3D7_1463500). PC transfer activity of the recombinant Fam-A proteins carrying a hexahistidine tag at their N termini was tested using a standard PC transfer assay (see the ‘Materials and Methods‘ section). **E**. Phosphatidylcholine (PC) transfer of full length Fam-a protein PBANKA_1327251 and a mutated form in which the final C-terminal alpha helix of the START domain has been deleted. In **D**. and **E**. the *P*. *falciparum* phospholipid transfer protein PFA0210c (PF3D7_0104200) was used as a positive control and a sample without Fam-A protein was used as negative control. White bars: control reactions; light grey bars: reactions with *P*. *berghei* proteins; dark grey bars: reactions with *P*. *chabaudi* proteins; black bars: reaction with *P*. *falciparum* protein. All reactions were set up in triplicate. The error bars indicate the standard deviation.

The expression of variant exported proteins in liver-stages indicates that these proteins promote parasite development not only in the blood but also in the liver either by supporting intracellular parasite development, or through the manipulation of the host immune response. By sequence analysis both PIR and Fam-B proteins do not contain domains with homology to known functional protein domains from other eukaryotes, including other Apicomplexan parasites, which would reveal their function in parasite growth inside RBC and hepatocytes. In contrast, Fam-A proteins are the only *Plasmodium* variant exported proteins that contain a domain with homology to a functional domain of proteins of other eukaryotes. This domain, the steroidogenic acute regulatory-related lipid transfer (START) domain, is involved in translocation of phospholipids, ceramide or fatty acids between membranes. The START domains represent the majority of these fam-A protein sequences. For example the START domain comprises 83% of the Fam-a protein PBANKA_1327251 and 96% of the FAM-a protein PCHAS_1331900. In **Fig 9** a schematic representation of the predicted tertiary structure of the START domain of Fam-a2 is shown, threaded against the resolved holotypic structure of the STAR-D2 domain in the human phosphatidylcholine transfer protein. In the *Plasmodium* START domains an expanded protein loop between β-sheets 7 and 8 is present (**Fig 9).**

As Fam-A proteins are expressed in liver stages, where the parasite requires host-derived phosphatidylcholine (PC) [41], we tested the ability of recombinant Fam-A proteins from *P*. *berghei* and *P*. *chabaudi* (and the single *P*. *falciparum* Fam-A) to transfer PC using a standard *in vitro* PC assay. In this assay protein-dependent transfer of radioactive PC from a small population of donor vesicles to a larger population of acceptor vesicles is measured. To capture potential differences between different Fam-A orthologues, we choose four *P*. *chabaudi* orthologues that were distally distributed on the Fam-A phylogenetic tree (see above), two *P*. *berghei* orthologues and the single *P*. *falciparum* Fam-A protein (**Fig 9D**). We expressed these as recombinant proteins containing a single hexahistidine tag at their N-terminus, which were purified over Ni2+ affinity resin and further purified by size exclusion chromatography. In the *in vitro* PC assay one of the four recombinant Fam-A proteins from *P*. *chabaudi* (PCHAS_1331900) and one of the two recombinant *P*. *berghei* proteins (PBANKA_1327251) showed robust PC transfer activity, whereas *P*. *falciparum* Fam-A reproducibly showed a lower, but detectable, level of PC transfer activity (**Fig 9D**). The activity level of the *P*. *chabaudi* and *P*. *berghei* proteins was identical to that of the previously described *P*. *falciparum* START-domain containing protein PFA0210c (PF3D7_0104200) and its orthologues in *Plasmodium knowlesi* and *P*. *chabaudi* [52]. The five other *P*. *chabaudi* and *P*. *berghei* Fam-A proteins did not show PC transfer activity, with activity levels comparable to the no protein control (**Fig 9D**). The activity of these Fam-A proteins (or lack thereof) was detected in multiple assays and was independent of the affinity tag as addition of an MBP tag (at the N terminus). In addition we have expressed and purified Fam-a protein PBANKA_132751 in which we have deleted the final C-terminal alpha helix of the START domain. This mutation is known to abolish the activity of START-domain-containing proteins [53] and used this mutated recombined protein to test PC transfer activity *in vitro* (Fig 9E). We found that PC transfer activity is significantly reduced of the mutated protein compared to the PC transfer activity of the full length protein, confirming the functionality of the START domain. It has previously been speculated that Fam-A proteins may be cholesterol transfer proteins based on the structural resemblance of the Fam-A proteins with MLN64, a human cholesterol transfer protein [35]. We tested the ability of two Fam-A proteins, PCHAS_120120 (which does not transfer PC), and PCHAS_1331900 (which does transfer PC), to transfer cholesterol using a standard cholesterol-binding assay. No cholesterol binding activity was detected for either protein (**S6 Fig**). Combined our results indicate that at least one *P*. *chabaudi* and one *P*. *berghei* Fam-A protein are phospholipid transfer proteins, whereas no evidence was found for cholesterol transfer activity.

## Discussion

Most studies of *Plasmodium* exported proteins encoded by multigene families have focussed on the *P*. *falciparum*-specific *var* family [1–6, 10, 54]. This family encodes variant proteins that are exposed on the outside of the iRBC surface membrane and have an essential role in sequestration of iRBC, as they bind directly to certain endothelial cell receptors [6, 19, 55–58]. Also for proteins of two other *P*. *falciparum*-specific multigene families, *stevor* and *rifin*, evidence for an iRBC surface membrane location has been reported and both are believed to mediate interactions between iRBC and uninfected RBC, resulting in rosetting [21, 23, 24, 59]. With the exception of *Plasmodium* species of the subgenus Laverania (such has *P*. *falciparum* and *P*. *reichnowi* [60]), other mammalian *Plasmodium* species lack genes with clear orthology to either the *var*, *stevor* or *rifin* multigene families. However, many other *Plasmodium* species contain a large multigene family encoding PIR proteins that are exported into the iRBC. These proteins have structural similarities with domains of *P*. *falciparum* RIFIN proteins [8, 21, 30, 35] and it has been suggested that PIRs are adhesins that enable parasites to rosette and sequester in the absence of the *va*r, *stevor* or *rifin* families [21, 32]. For *P*. *vivax* PIR proteins some evidence has been presented for a role in iRBC sequestration [9, 31] and a subset of recombinant *P*. *chabaudi* PIRs showed binding to mouse RBC suggesting a role for these proteins in rosette formation and/or invasion [32]. PIR proteins are, however, also expressed in *Plasmodium* species that lack sequestration of iRBC, such as *P*. *yoelii*, and PIRs are abundantly expressed in RMP blood stages that do not sequester, for example (young) trophozoites and gametocytes of *P*. *berghei* [33, 36]. These observations suggest that *Plasmodium* PIRs are not exclusively involved in iRBC sequestration and/or rosetting. Our observations on the cytosolic location of most tagged family members and their absence from the outer host-cell surface membrane also indicate that these members are not involved in direct interactions between iRBC and host cells that lead to sequestration or rosetting.

Several features of the RMP multigene families suggest that different members of these families may fulfil different functions during blood stage development. The presence of structurally different phylogenetic clades that are conserved between different RMP suggest functional diversification among family members ([8, 33] and this study). In addition, differences in timing of expression and cellular location between family members may indicate functional diversification ([33, 36] and this study). However, it is possible that the different members share the same function but do so at a different cellular location or at a different time point during development. Therefore, the conserved, structural differences between family members might not be related to separate functions but rather linked to differences in stage-specific expression, trafficking and location.

The expression of multigene family members in liver-stages also calls into question their exclusive role in iRBC sequestration and rosetting or in other processes related to remodelling of the iRBC. Although we have tagged only a limited number of members of the three families, we have found so far no evidence that members of different phylogenetic clades are specifically expressed either in liver- or in blood-stages. Indeed seven out of eight family members that were expressed in blood-stages were also expressed in liver-stages.

In the absence of a known function for members of the three families in iRBC we can only speculate on their role in liver-stages. For example, these proteins may facilitate an efficient formation of mature schizonts and daughter merozoites in the liver (see below) or they could help establish and/or promote a subsequent blood infection just after the infected hepatocyte ruptures and discharges the protein contents of its PV in the blood in addition to the release of merozoites.

As many of the multigene family members appear not to be located at the outer side of the iRBC surface membrane, the immune pressure on these variant proteins is likely not due to their detection on the surface of iRBC and removal of iRBC by macrophages in the spleen as has been suggested for iRBC surface-exposed proteins [18–20]. Interaction with the immune system will mainly occur when these protein complexes are released into the blood after host cell rupture. It has been shown that changes in PIR repertoire expression correlates with differences in virulence of *P*. *chabaudi*. Blood stage populations with a limited PIR expression were more virulent than blood stage populations with a larger repertoire of expressed PIRs. Mosquito transmission promoted a generalized increase in PIR expression in the parasite population that infected RBCs [45]. These observations may indicate that PIR expression influences immune responses, which may affect parasite survival and virulence. Since the liver has a unique local immune system with systemic impact [61], it is conceivable that the release of parasite proteins after rupture of the PVM and subsequent merozoite release impact on innate and adaptive immune responses that later may influence blood-stage infections.

All examined proteins were exported into the PV of the liver-stages, apart from one PIR protein. However, unlike in blood-stages, we did not find evidence for transport of these proteins across the PVM, suggesting that these proteins are not involved in either remodelling the host hepatocyte or transport parasite or hepatocyte factors through the hepatocyte cytoplasm. For two Fam-a members we provide evidence for a location on/at the PVM. In the PVM of blood-stages the translocon of exported proteins (PTEX) is implicated in the translocation of parasite proteins from the PV into the iRBC cytoplasm [3, 48, 62]. Interestingly, two recent studies provide evidence that a putative PTEX in the PVM of liver stages is different from those in the PVM of blood stages [63, 64]. Specifically, one of the main components, HSP101, is not expressed in liver-stages. HSP101 is a member of the ClpA/B chaperone family and is proposed to unfold cargo proteins [65]. Thus a different PTEX composition (or the absence of a functional translocon) might be responsible for the retention of blood-stage exported proteins in the PV of liver stages [63, 64]. However, it remains possible that the members of the multigene families are exported into the hepatocyte cytoplasm but below the detection limit or that the fluorescent-tag hampered the efficient passage of proteins through the PVM. This latter explanation is less likely, since most tagged proteins were able to cross the PVM of blood-stages ([36]; this study).

For several family members we found that the mCherry-tagged protein was expressed in liver stages, whereas no or weaker expression was detected when the same member was tagged with GFP. This may suggest that the GFP-tag hampered expression or transport of the protein to the correct location, resulting in protein degradation or may be explained by differences in specific properties of GFP and mCherry that influence fluorescence intensity, i.e. the higher pH sensitivity of GFP compared to mCherry [66–68]. However, the lack of expression of the GFP-tagged version might also be due to chance, i.e. that the encoding gene had not been switched on in the liver on this occasion as the expression switching rate of family members is relatively frequent. This is supported by the observation of the presence of both the GFP- and the mCherry-tagged version of the IBIS protein in the PV of liver stages, a protein encoded by a single copy gene that is stably expressed in all parasites.

In blood-stages the IBIS protein is found in discrete membranous structures in the iRBC cytoplasm that exhibits characteristics of *P*. *falciparum* Maurer’s clefts and its location in the PV of liver-stages had previously been demonstrated [47]. The SMAC protein, the other tagged protein that is encoded by a single copy gene, is also located in the iRBC cytoplasm and has been implicated in the transport of proteins that facilitate CD36-mediated sequestration of *P*. *berghei* schizonts [16]. It is therefore possible that IBIS and SMAC are both components of larger protein complexes in the iRBC cytoplasm, protein complexes which may also interact with members of the three multigene families. Such protein complexes may fulfil similar functions in the iRBC cytoplasm and in the PV of liver-stages. For example, these complexes may be involved in transport of factors essential for schizont maturation and merozoite formation.

Both PIR and Fam-B proteins do not contain domains that have homology to functional domains of proteins from other eukaryotes including proteins of other apicomplexan parasites that would point to a function for parasite growth within RBCs or hepatocytes. In contrast, Fam-A proteins contain a START (StAR-related lipid-transfer) domain with homology to a functional domain of StAR (Steroidogenic Acute Regulatory) proteins in other eukaryotes, so named based on their capacity to transport a large variety of lipids and sterols between membranes [35, 69–71]. The START domain is a module of about 210 residues, which binds lipids such as glycerolipids, sphingolipids and sterols. We show that several Fam-A proteins have robust phosphatidylcholine (PC) transfer activity *in vitro*. This activity was in the same range as previously described for the exported *P*. *falciparum* phospholipid transfer protein PF3D7_0104200 that also contains a START domain [52]. Such PC transfer activity is in support of a role of these proteins in parasite development within the host cell. Uptake of host cell PC has recently been shown to be key for malaria liver stage development [41] and the presence of Fam-A proteins with PC transfer activity in the PV may indicate that they are involved in uptake and transfer of PC from the hepatocyte. PC is also a main component of membranes of *Plasmodium* blood stages and the expression of Fam-A members in the cytoplasm of iRBC may further designate their role in uptake of host PC. However, these proteins may also be involved in transfer of parasite-derived phosphatidylcholine into the host cell for the assembly of membrane-bound compartments in iRBC, as has been suggested for the *P*. *falciparum* START-domain containing protein PF3D7_0104200 [52]. *Plasmodium* parasites have different enzymatic pathways for the *de novo*-synthesis of PC [72]. Interestingly, rodent and non-rodent *Plasmodium* species differ in their phospholipid metabolic pathways [72, 73]. Non-rodent *Plasmodium* species have an additional PC synthesis pathway, a plant-like pathway that relies on serine decarboxylase and phosphoethanolamine N-methyltransferase activities, which diverts host serine to provide additional PC and phosphatidylethanolamine to the parasite. The absence of this pathway in rodent malaria parasites may explain why the rodent parasites have an expanded family of START-domain containing proteins compared to non-rodent *Plasmodium* species. In the absence of this *de novo* synthesis pathway rodent *Plasmodium* species may be (more) reliant on uptake of host PC for synthesis of their membranes during growth and multiplication. It is also conceivable that rodent parasites owe their very short developmental cycles (24 and 48 hour cycle of blood- and liver-stages, respectively) to a higher and more efficient uptake of host lipids for membrane synthesis during intracellular growth. It is interesting to note that Fam-A members were not only localized in the iRBC cytoplasm but also at the surface membrane which may suggest that these proteins may be involved in uptake of host lipids from the serum and subsequent transfer into the parasite.

It is known that also hepatocyte-derived cholesterol is transported to the parasitophorous vacuole of liver stages [74, 75] and that blood stages scavenge host cholesterol since *Plasmodium* parasites cannot synthesize cholesterol [76, 77]. It has been speculated that the Fam-A proteins may be cholesterol transfer proteins based on the structural resemblance of the Fam-A proteins with MLN64, a cholesterol transfer protein [35]. We found no evidence of cholesterol transfer activity of the few Fam-A proteins we have tested. It is however possible that other Fam-A members play a role in transport in lipids other than PC, including cholesterol. In other eukaryotes START domain proteins have been divided into distinct subfamilies, each subfamily being more specialized in the transport and/or sensing of specific lipid ligand species and START domain containing proteins act in a variety of distinct physiological processes, such as lipid transfer between intracellular compartments, lipid metabolism and modulation of signaling events [70, 71].

Combined, our observations indicate that (a subset of) Fam-A proteins are involved in the transfer of PC. Transfer of PC may either be from the host cell into the parasite for membrane synthesis during intracellular growth or may be of parasite-derived PC onto the PV/PVM and into the host cell cytoplasm to form trafficking networks contiguous with the PV/PVM. This PC transfer activity is the first demonstration of a biological function of any exported variant protein family of rodent malaria parasites and further exploration of capacity of different members of Fam-A family to transfer specific/different lipid moieties transfer is required to better understand the role of host nutrient uptake and intracellular parasite development. Our observations on liver stage expression expand the role for these proteins beyond their role in iRBC interactions with host cells, such as sequestration or rosetting. Our observations of proteins of RMP multigene families may also be of relevance for understanding the role of multigene families, other than the *var* family, in human malaria. PIR proteins are also expressed by *P*. *vivax* and *P*. *knowlesi* and several features of PIRs are shared with *P*. *falciparum* RIFIN and STEVOR proteins [10, 21].

## Materials and Methods

### Improvement of the *P*. *berghei* genome assembly

Using *P*. *berghei* ANKA (cl15cy1) genomic DNA we generated a C2P4 library of fragments with a mean length of 8kb. By Pacific Biosciences sequencing 20 SMRT cells were generated (Accession number ERS531640), which were assembled with the HGAP assembler using the default parameter [40]. The resulting 61 contigs were ordered using ABACAS (Algorithm-Based Automatic Contiguation of Assembled Sequences; [78]) against the current *P*. *berghei* ANKA (cl15cy1) reference genome (version2; [33]). Contigs that contained mouse DNA contamination or contained incorrect assemblies (for example chimeras) were excluded. This resulted in the assembly of 14 chromosomes, two plastid genomes and a remaining 11 unassigned contigs in the bin). Contigs were corrected for frameshift with ICORN (Iterative Correction Of Reference Nucleotides; [79]). The improved sequence was annotated through RATT (Rapid Annotation Transfer Tool; [80]) and ab initio gene finding [81] and combined as described in [33]. The annotation was further manually improved.

### Phylogenetic analyses

Fam-a: All homologous sequences were extracted from PlasmoDB (version 12.0) after BLAST analysis and from GeneDB for the re-annotated sequences. Initial alignment of coding sequences established that structural variation was relatively low and therefore, to maximise resolution, the phylogenetic analysis would be carried out on nucleotide sequences containing both exons and introns. Multiple sequence alignment was carried out with ClustalW [82] within the BioEedit suite [83], and then manually adjusted. The final alignment contains 1328 characters; due to substantial length variation at the 5’ end of the genes, the first exon, first intron and a portion of the second exon were omitted because they do not align. The data set includes 313 taxa; nine *P*. *berghei* sequences and 22 *P*. *yoelli* 17X sequences were omitted because they failed to align satisfactorily. Phylogenetic analysis was carried using two methods; maximum likelihood (ML) using RAXML v8.0 [84] and Bayesian Inference (BI) using MrBayes v3.2.3 [85]. In both cases, a general time-reversible model with a correction for rate heterogeneity (GTR+Γ) was applied. Node robustness was assessed in the ML analysis through 100 non-parametric bootstraps. The Bayesian analysis was carried out with four independent Monte Carlo Markov Chain (MCMC) chains, each running for 5 million generations, with a sampling frequency of 1000 generations and a burn-in of 10%. The potential scale reduction factor (PSRF) for tree length approached one (1.000074), indicating that the MCMC analysis reached convergence. Analysis with Tracer [86] confirmed that the effective sample sizes for both log-Likelihood (ESS = 1128) and tree length (ESS = 18630) were sufficient for convergence. To assess the effect of dynamic differences in substitution rate across the genome, a second, partitioned Bayesian analysis was carried out on the same alignment in which each exon and intron was independently modelled.

Fam-b: All homologous sequences were extracted from PlasmoDB (version 12.0) and from GeneDB for the re-annotated sequences and aligned as described above. Initial alignment again established that, to maximise resolution, the phylogenetic analysis should be carried out on nucleotide sequences containing both exons and the single intron. The final alignment contains 1082 characters. The data set includes 120 taxa; nine *P*. *berghei* sequences, two *P*. *chabaudi* sequences and 13 *P*. *yoelli* 17X sequences were omitted because they failed to align satisfactorily. ML and BI phylogenetic analyses were carried out as described above. In the Bayesian analysis without partitioning, the MCMC analysis failed to reach convergence, (PSRF for tree length = 1.028711); effective sample sizes for both log-Likelihood (ESS = 23) and tree length (ESS = 89) were substantially less than 100. A second, partitioned Bayesian analysis also failed to reach convergence (PSRF = 1.080465; log-Likelihood ESS = 4; tree length ESS = 18). We attempted to produce convergence in the MCMC runs by constraining each with a starting tree topology estimated using maximum parsimony in MEGA v6 [87]. However, this analysis also failed to achieve convergence in log-Likelihood after 5 million generations (PSRF = 1.005; log-Likelihood ESS = 20; tree length ESS = 1865). Hence, it seems ambiguity in these data make the estimate inherently unstable. In light of this, our BI estimate is based on a single MCMC run from the MP-primed analysis that did converge (log-Likelihood ESS = 731; tree length ESS = 3154).

RNA-seq data: RNA-seq data of the three multigene families from RMP blood-stages was obtained from Otto et al. [33]. For *P*. *berghei fam-a*, *fam-b* and *pir* families the RNAseq reads were remapped to the improved *P*. *berghei* genome as described [33]. Transcript abundance is expressed in FPKM (fragments per kilo base of exon per million fragments mapped). FPKM cut-off values were calculated for each RMP as described [33]. Expression evidence was defined as FPKM values >21 for *P*. *berghei*, >11 for *P*. *chabaudi* and >11 for *P*. *yoelii*. We defined the following classes for the level of expression for the *fam-a* and *fam-b* genes: class 1: less than twice the cut-off values; class 2: between 2 and 4 times the cut-off levels; class 3: between 4 and 8 times the cut-off levels; class 4: >8 times the cut-off levels. Due to the improved *P*. *berghei* genome sequence (see above), several collapsed repeats were now separated in the assembly, resulting in base perfect duplicated copies of the *fam-a*, *fam-b* and *pir* genes. As default, the expression level (FPKM value) of these duplicated genes is zero. We estimated the amount of unique (non-repetitive) base pairs of all genes of one family by blasting all genes against themselves and subtracting from the top blast hit, the length (bp) of the overlap of the total gene length (bp) of the gene (only for those genes that show an identity >98.5%).

### Experimental animals and (reference) *P*. *berghei* lines

Female Swiss OF1 mice (6–8 weeks; Charles River, F) and female Wistar and Brown Norway rats (7 weeks, Charles River, F) were used. Three reference ‘wild type’ *P*. *berghei* ANKA parasite lines were used: i) line cl15cy1 (ANKAwt) [46] ii) reporter line 1037cl1 (ANKA-GFP-Luc_schiz_; mutant RMgm-32; www.pberghei.eu) which contains the fusion gene *gfp-luc* gene under control of the schizont-specific *ama1* promoter integrated into the silent *230p* gene locus (PBANKA_030600) and does not contain a drug-selectable marker [79] and iii) reporter line 676m1cl1 (ANKA-GFP-Luc_con_; mutant RMgm-29; www.pberghei.eu) which contains the fusion gene *gfp-luc* gene under control of the constitutive *eef1α* promoter integrated into the silent *230p* gene locus (PBANKA_030600) and does not contain a drug-selectable marker [88].

Ethics statement: All animal experiments of this study were approved by the Animal Experiments Committee of the Leiden University Medical Center (DEC 07171; DEC 10099; DEC12042). The Dutch Experiments on Animal Act is established under European guidelines (EU directive no. 86/609/EEC regarding the Protection of Animals used for Experimental and Other Scientific Purposes).

### Generation, selection and genotype analysis of transgenic parasites expressing fluorescently-tagged proteins

To generate transgenic parasites expressing C-terminally tagged mCherry proteins, construct pL1419 was used [16]. The *smac* targeting region was then replaced by a targeting region of the candidate genes listed in supplemental S2 Table. For pL2067 and pl2069 (double cross-over recombination plasmids), a second targeting region was introduced (HindIII/ApaI). Construct pL1817 was used to generate transgenic parasites expressing C-terminally tagged GFP proteins [36]. To create C-terminally tagged fluorescent plasmid that contain the human *dhfr/ts* selectable marker, the *T*. *gondii* dihydrofolate reductase/thymidylate synthase (*dhfr/ts*) selectable cassette was exchanged for the h *dhfr/ts* selectable cassette of pL0006 (HindIII/EcoRV; MR4, http://www.mr4.org). Details of the primers, DNA constructs and the genotype analysis of all mutants have been submitted to the database of genetically modified rodent malaria parasites (RMgmDB, www.pberghei.eu). For the generation of parasite expressing two C-terminally tagged fluorescent proteins (mCherry and GFP), parasites were first transfected with a DNA construct that contains the *dhfr/ts* and targets the first gene. These transfected parasites were selected with pyrimethamine. Subsequently these transgenic parasites were transfected with a DNA construct that contains the human *dhfr/ts* selectable marker and targets the second gene. These transfected parasites were selected with pyrimethamine WR99210 [48]. Transfection of *P*. *berghei* parasites with linearized plasmids, selection and cloning of transgenic and mutant parasite lines was performed as described [46]. Correct integration of the DNA constructs was determined by diagnostic PCR and Southern analysis of chromosomes separated by pulse-field gel (PFG) electrophoresis. Southern blots were hybridized with the following probes: 3’UTR *dhfr/ts* of *P*. *berghei ANKA* and a mixed probe human *dhfr* [89]; ~800bp fragment of 5’UTR of PBANKA_0508000 located on chromosome 5 (primer set: 4100 5’- GGGGTACCGCACATCTACAAATTGCATGTC and 4101 5’- CCCAAGCTTTTGAACCAGTTACAGGCTTG).

### Analysis of blood stage development of transgenic parasites

For analysis of transgene expression in blood stages, most parasites were collected from asynchronous blood stage infections in Swiss mice. These mice were either infected intravenously with single infected red blood cell (during the cloning procedure) or intraperitoneally (i.p.) with 10^5^ infected red blood cells. To monitor parasite development in Brown Norway, rats were injected i.p. with 10^5^ parasites. Parasitemia in rats was monitored by analysis of Giemsa-stained thin smears of tail blood collected during the course of infection to a maximum of 51 days post infection. To collect parasites for FACS analysis of fluorescence (see below) 50μ of tail blood of rats was used to infect 2 mice. At a parasitemia of 1–3% tail blood was collected from these mice for FACS analysis.

### Analysis of expression of fluorescently-tagged proteins in blood stages of transgenic parasites

For analysis of mCherry and/or GFP expression of the transgenic lines, tail blood of infected mice or infected erythrocytes from *in vitro* cultures [36] were collected in PBS or complete 1640-RPMI culture medium and were examined by FACS (see below) or fluorescence microscopy using a Leica DMR fluorescent microscope with standard GFP and Texas Red filters. Parasites nuclei were labelled by staining with Hoechst-33258 (Sigma, NL) and red blood cell surface membranes were stained with the anti-mouse TER-119-FITC labelled antibody (eBioscience, NL). Briefly, erythrocytes were stained with TER-119-FITC antibody (1:200) and Hoechst-33258 (2μmol/L) at room temperature (RT) for 30 min and washed with 500μL of RPMI-1640 medium (400*g*; 2min). For DNA visualization, Hoechst-33258 (2μmol/L) was added during the incubation of the secondary antibody. Pelleted cells (400*g*, 2min) were resuspended in RPMI-1640 medium.

The percentage of blood stage parasites that express mCherry and/or GFP was determined by FACS analysis of cultured blood stages. In brief, infected tail blood (10 μL) with a parasitemia between 1 and 3% was cultured overnight in 1ml complete RPMI1640 culture medium at 37°C under standard conditions for the culture of *P*. *berghei* blood stage [46]. Cultured blood samples were then collected and stained with Hoechst-33258 (2 μmol/L, Sigma, NL) for 1 hr at 37°C in the dark and analysed using a FACScan (BD LSR II, Becton Dickinson, CA, USA) with filter 440/40 for Hoechst signals and filter 610/20 for mCherry fluorescence. For FACS analysis the population of mature schizonts was selected on their Hoechst-fluorescence intensity and the percentage of mCherry-expressing parasites was calculated by dividing the number of mCherry-positive schizonts by the total number of mature schizonts [16].

Statistical analyses were performed using Student’s t-test and Two-way ANOVA with the GraphPad Prism software package 5 (GraphPad Software, Inc).

### Analysis of expression of fluorescently-tagged proteins in mosquito- and liver-stages of transgenic parasites

Feeding of *A*. *stephensi* mosquitoes and determination of oocyst production was performed as described [90]. Oocyst infection and expression of fluorescently-tagged proteins were monitored between day 8 and 12 post mosquito infection. *P*. *berghei* sporozoites were isolated from salivary glands of infected *Anopheles stephensi* mosquitoes 18–24 days after an infectious blood meal. Fluorescence in oocysts and sporozoites was analsysed using a Leica MZ16 FA microscope.

The human hepatocyte carcinoma cell line Huh7 (JCRB0403, JCRB Cell Bank, JP) is used for *in vitro* cultures of the liver stages. Isolated sporozoites (5×10^4^) were added to monolayers of Huh7 cells on coverslips in 24 well plates (with a confluency of 80–90%) in ‘complete’ DMEM [90]. At different time points after infection (30, 44 and 48hpi), nuclei were stained with 1 μg/ml Hoechst 33342 and live imaging of the different liver stages, GFP and or mCherry-expressing Huh7 was performed using a DM RA Leica fluorescence microscope.

For immunofluorescence analysis of liver stages, 5×10^4^ sporozoites were added to a monolayer of Huh7 cells on coverslips in 24 well plates in ‘complete’ RPMI 1640 medium supplemented with 10% (vol/vol) fetal bovine serum (FBS), 2% (vol/vol) penicillin-streptomycin, 1% (vol/vol) GlutaMAX (Invitrogen), and maintained at 37°C with 5% CO_2_. At 30, 44 and 48 hr after infection, cells were fixed with 4% paraformaldehyde, permeabilized with 0.5% Triton-X 100 in PBS, blocked with 10% FBS in PBS, and subsequently stained with primary and secondary antibodies overnight at 4°Cand for 1h, respectively. Primary antibodies used were anti-GFP (594 Alexa Fluor # A21209; Life-Technologies) anti-mCherry (DsRed polyclonal 632496; Clontech and monoclonal # M11217; Life-Technologies). Secondary antibodies used were anti-mouse conjugated to Alexa Fluor 488 (# A11029 Life-Technologies) for GFP and respectively anti-rabbit conjugated to Alexa Fluor 594 (A-21207; Invitrogen) and anti-GFP mouse IgG1k (# 11814460001; Roche) for mCherry. Additionally parasites were stained with primary antibodies: anti-PbEXP1 (PBANKA_092670) raised in chicken [91] or with anti-rabbit PbUIS4 (PBANKA_092670; [92]) and anti-chicken or anti-rabbit secondary antibodies, conjugated to Alexa Fluor® 488 were used for immune-detection (Invitrogen).

Nuclei were stained with Hoechst-33342. Cells were mounted in Image-iT FX Signal Enhancer (Molecular Probes) and examined using a DM RA or a TCS SP8 Leica fluorescence microscope. Images analysis was done with the Leica LAS X software.

For analysis of blood infections after sporozoite infection, Swiss mice were inoculated with 1x10^4^sporozoites by intravenous injection. Blood stage infections were monitored by analysis of Giemsa-stained thin smears of tail blood collected on day 5–8 after inoculation of sporozoites. Infected tail blood was collected at a parasitemia of 1–3%.

### Structural analysis and cloning of fam-a genes and protein purification

A structural model of *P*. *berghei* Fam-a gene (PBANKA_1327251) was generated by threading against the resolved structure of the STAR-D2 domain in the human Phosphatidylcholine Transfer Protein (1LN1; [93]) using Phyre2 [94].

Sequences encoding MLN64 (human isoform 2) and the *Plasmodium* Fam-A family members tested were synthesized (GeneArt and IDT) without introns and codon-optimized for expression in *Escherichia coli*. The region encoding the START domain was amplified using the primers listed in **S3 Table** and the resulting DNA fragment was cloned into pMALc2x. Additionally, for the Fam-a protein PBANKA_1327251, we generated a recombinant protein that lacks the final C-terminal alpha helix of the START domain. In the case of MLN64, the restriction sites were included in the synthesized DNA and this was cloned directly into pMALc2x. The sequences of the resulting plasmids were verified by sequencing (Source Biosciences). The plasmids were subsequently transformed in the *E*. *coli* strain BL21(DE3) and expression of hexahistidine-tagged Fam A protein was induced in 200 ml bacterial cultures by the addition of isopropyl β-D-1-thiogalactopyranoside (IPTG) to 0.5 mM when the culture was at an OD600 of ~0.5. The bacteria were harvested after an overnight incubation at 18°C and resuspended in column buffer (20 mM Tris, pH 7.4, 500 mM NaCl, 20 mM imidazole) containing protease inhibitors (Complete EDTA-free Cocktail, Roche). The bacteria were lysed with a cell disruptor (Constant Cell Disruption Systems) and the lysate sonicated with a microtip for three 30-second pulses (50% duty cycle, setting 4; Vibracell, Sonics and Materials). The lysate was clarified by centrifugation in a JA25.5 rotor at 9000 x g for thirty minutes. The clarified lysate was mixed with Ni^2+^ resin (Qiagen) and incubated at 4°C for one hour while rotating. The mixture was poured into a 1.5 x 12 cm chromatography column (BioRad) and the resin was washed with 50 column volumes of column buffer. The protein was eluted with five column volumes of column buffer supplemented with 250 mM imidazole. The eluate was concentrated to 0.5–1.5 ml using a Vivaspin 15 concentrator with a molecular weight cut-off of 10,000 MW (Sartorium Stedim Biotech) and loaded onto a HiLoad 26/60 Superdex 200 prep-grade column equilibrated in standard assay buffer (10 mM HEPES-Na^+^, pH 7.4, 1 mM EDTA, 50 mM NaCl, pH7.4). Elution of protein was detected through monitoring the UV absorption of the eluate, followed by SDS-PAGE. The fractions containing monomeric protein were concentrated as described above, aliquoted and snap frozen in liquid nitrogen. The MBP-PFA0210c-His6 fusion used as positive control was produced as described previously [52].

### Phospholipid transfer assay

The phospholipid transfer activity of the Fam A proteins was measured as previously described [95]. Briefly, acceptor vesicles were produced by mixing phosphatidylcholine and phosphatidic acid in a molar ratio of 98:2, whereas donor vesicles were produced by mixing phosphatidylcholine, phosphatidic acid, N-lactosyl-phosphatidylethanolamine (all non-radioactive lipids were obtained from Avanti Polar Lipids, Inc. and were dissolved in chloroform) in a molar ratio of 88:2:10, with a trace of ^14^C-labeled phosphatidylcholine (L-α-DiPalmitoyl-Phosphatidylcholine; Perkin Elmer). The phospholipid mixtures were supplemented with 200 μl chloroform, dried under a stream of N_2_ gas until completely dry and then resuspended in standard assay buffer (10 mM HEPES, pH 7.4, 1 mM EDTA and 50 mM NaCl) such that the total concentration of phospholipid was 2.3 mM. This mixture was sonicated in a sonicating water bath (Ultrawave U300H) until the solution became completely translucent. Transfer assays were set up by mixing 30 μl acceptor vesicles (69 nM total phospholipid), 10 μl donor vesicles (23 nM total phospholipid) and 5 μl 20 mg/ml essentially fatty-acid free bovine serum albumin (1 mg/ml final concentration; Sigma), followed by the addition of Fam A or control protein to a final concentration of 25 μg/ml and standard assay buffer to bring the reaction volume to 100 μl. This mixture was incubated at 37°C for thirty minutes. A 5 μl aliquot was removed to measure total radioactivity in the sample using scintillation counting. To the remainder, 37.5 μl of a 400 μg/ml solution of agglutinin RCA120 (lectin from *Ricinus communis*; Sigma) in standard assay buffer was added (to a final concentration of 110 μg/ml) to agglutinate the donor vesicles and the samples were incubated on ice for thirty minutes and then at room temperature for ten minutes. The agglutinated donor vesicles were removed by centrifugation for six minutes at 13,000 rpm in a microcentrifuge. The radioactivity in the supernatant, which represents the amount of radioactive phospholipid that was transferred to the acceptor vesicles, was then measured using scintillation counting and the amount of transfer of the radioactivity was calculated.

### Cholesterol binding assay

The fluorescent cholesterol reporter NBD-cholesterol (22-(N-(7-Nitrobenz-2-Oxa-1,3-Diazol-4-yl)Amino)-23,24-Bisnor-5-Cholen-3β-Ol) (Life Technologies) was used to verify cholesterol binding. The sterol was added to a final concentration of 600 nM in a Suprasil quartz fluorescence cuvette (path length 3x3mm) (Hellma) pre-incubated at 37°C, containing 200 μl of 25 mM potassium phosphate buffer (pH 7.4) with 5% DMSO, and mixed. Protein was titrated into the cuvette and the fluorescence was recorded every 0.5 seconds for a 5 min period. The measurements were performed using an FP-6300 spectrofluorometer (JASCO). NBD-cholesterol was excited at 473 nm and fluorescence emission was monitored at 530 nm, at high intensity.

## Supporting Information

S1 Fig The fold up- and down regulation of transcription of members of the *fam-a*, *fam-b* and *pir* multigene families in blood stages of two cloned *P*. *berghei* ANKA reference lines (line1, 2) (ring, red; trophozoite, green; schizont, purple; gametocyte, black). The fold up- or down-regulation of expressed genes (RNAseq FPKM values >21; S1 Table) is calculated by dividing the FPKM value observed for each gene for a particular blood stage by the FPKM value for the same gene and blood stage of the other line. Dotted lines show a 1.5 fold up- or down regulation. Arrows indicated genes selected for tagging.
(PDF)

S2 Fig Analysis of blood stages expressing mCherry-tagged (non-exported) proteins with a predominant localization in the cytoplasm or in organelles of the parasite. **A**. Location of mCherry-tagged PBANKA_0519300 (line 2086) in the cytoplasm of trophozoites and schizonts. **B**. Location of mCherry-tagged PBANKA_1400600 (line 1915) with an organellar (rhoptry‐like) localization in merozoites. The plasma membrane of the red blood cell is stained with TER119 antibodies (green) and parasite nuclei are stained with Hoechst. Troph: trophozoite; Sch: schizont and Mz: merozoroite. Scale bar: 2μm.
(PDF)

S3 Fig Genotyping of clones of different transgenic lines by Southern analysis of pulsed field gel (PFG) separated chromosomes. Separated chromosomes were hybridized with a probe recognizing the 3’UTR of the bifunctional *dihydrofolate reductase-thymidylate synthase* (*dhfr/ts*) gene of *P*. *berghei*. This probe recognises the endogenous 3’UTR of the *dhfr/ts* located on chromosome 7 and the 3’UTR of the integrated construct into the target gene for tagging with mCherry or GFP.
(PDF)

S4 Fig Percentage of fluorescent-positive schizonts (right panels) of cloned transgenic parasites expressing fluorescently tagged *fam-a* (**A**), *fam-b* (**B**) and *pir* (**C**) members during long-term infections in Brown Norway rats (2 rats per line R0 and R1 for Fam-a1 and PIR1; 1 rat for Fam-b1 and Fam-b2). In the left panels the course of parasitemia is shown in the rats. **D.** The course of parasitemia in rats infected with of a reference *P*. *berghei* ANKA line. **: p = 0.0062 (Two-way ANOVA).
(PDF)

S5 Fig Confocal microscopy analysis of the location of two Fam-a members in infected liver cells. Huh7 cells were infected with sporozoites of transgenic lines expressing either mCherry-tagged Fam-a1 or mCherry-tagged Fam-a2, fixed at 44 hpi and stained with antisera against two PVM-resident proteins (**A**. EXP1; **B**. IUS4; green) and with anti-mCherry antibodies (red). Fluorescence intensities for each fluorochrome were measured along the white line shown in the overlay image and plotted as distance versus intensity. Peaks of mCherry-staining overlap with both EXP1 and UIS4 staining. Nuclei are stained with Hoechst-33342 (blue). Scale bar: 2.5 μm, except for A lower panel, 10μm.
(PDF)

S6 Fig Cholesterol binding of three *P*. *chabaudi* Fam-A proteins. The binding of cholesterol by the recombinant Fam-A proteins PCHAS_1201200 and PCHAS_1331900 was tested by adding increasing amounts of protein to a solution containing 600 nM NBD-cholesterol. The emission of the fluorophore increases when it moves from the hydrophilic environment of the aqueous solvent to the hydrophobic environment of the binding pocket of the START. Hence an increase in amount of light emitted from the fluorophore indicates binding of the NBD-cholesterol to the START domain. In this case, no increase in emission was detected upon addition of the PCHAS_1201200, PCHAS_1331900 or the negative control, diubiquitin fused to a hexahistidine tag. Addition of the positive control protein MLN64 (also fused at its N terminus to a hexahistidine tag), lead to a steady, concentration-dependent increase in fluorescence emission, indicative of cholesterol binding.
(PDF)

S1 Table RNA-seq data (FPKM values) of rodent malaria parasites. **(1)** RNA-seq data (FPKM values) of fam-a and fam-b family members in different life cycle stages of *P*. *berghei* ANKA (PbA). **(2)**: RNA-seq data (FPKM values) of fam-a and fam-b family members in late trophozoite stage of *P*. *chabaudi* AS (PcAS; obtained from 4 different mice (Pc_M1-4). **(3)** RNA-seq data (FPKM values) of fam-a and fam-b family members in mixed blood stages stages of *P*. *yoelii* YM (PyYM) obtained from wild type (WT) parasites and the mutant PY01365-KO line. **(4):** RNA-seq data (FPKM values) of fam-a and fam-b family members in different life cycle stages of *P*. *berghei* ANKA (PbA) and Difference Class analysis. **(5)**: RNA-seq data (FPKM values) of pir family members in different life cycle stages of *P*. *berghei* ANKA (PbA). **(6):** RNA-seq data (FPKM values equal or above 21) of family members in different life cycle stages of *P*. *berghei* ANKA (PbA) presented in Fig 4C.
(XLSX)

S2 Table Detailed of selected *P*. *berghei* proteins for functional analysis by tagging. (XLSX)

S3 Table Primers used for making Fam A expression plasmids. (XLSX)
