# Correction: Variant Exported Blood-Stage Proteins Encoded by *Plasmodium* Multigene Families Are Expressed in Liver Stages Where They Are Exported into the Parasitophorous Vacuole

**DOI:** 10.1371/journal.ppat.1006107

**Published:** 2016-12-14

**Authors:** Aurélie Fougère, Andrew P. Jackson, Dafni Paraskevi Bechtsi, Joanna A. M. Braks, Takeshi Annoura, Jannik Fonager, Roberta Spaccapelo, Jai Ramesar, Séverine Chevalley-Maurel, Onny Klop, Annelies M. A. van der Laan, Hans J. Tanke, Clemens H. M. Kocken, Erica M. Pasini, Shahid M. Khan, Ulrike Böhme, Christiaan van Ooij, Thomas D. Otto, Chris J. Janse, Blandine Franke-Fayard

The third author's middle name is incorrectly listed as a last name. The correct name is: Dafni Paraskevi Bechtsi. The correct citation is: Fougère A, Jackson AP, Bechtsi DP, Braks JAM, Annoura T, Fonager J, et al. (2016) Variant Exported Blood-Stage Proteins Encoded by *Plasmodium* Multigene Families Are Expressed in Liver Stages Where They Are Exported into the Parasitophorous Vacuole. PLoS Pathog 12(11): e1005917. doi:10.1371/journal.ppat.1005917
