# Correction: Variant Exported Blood-Stage Proteins Encoded by *Plasmodium* Multigene Families Are Expressed in Liver Stages Where They Are Exported into the Parasitophorous Vacuole

**DOI:** 10.1371/journal.ppat.1006128

**Published:** 2017-01-17

**Authors:** Aurélie Fougère, Andrew P. Jackson, Dafni Paraskevi Bechtsi, Joanna A. M. Braks, Takeshi Annoura, Jannik Fonager, Roberta Spaccapelo, Jai Ramesar, Séverine Chevalley-Maurel, Onny Klop, Annelies M. A. van der Laan, Hans J. Tanke, Clemens H. M. Kocken, Erica M. Pasini, Shahid M. Khan, Ulrike Böhme, Christiaan van Ooij, Thomas D. Otto, Chris J. Janse, Blandine Franke-Fayard

There are errors in this article that the authors and publisher wish to correct. In Table 2, there are formatting errors in the table headings and in the “Double gene-tagging mutants (multigene families)” section. Please see the corrected Table 2 here. The publisher apologizes for the errors. In addition, in preparation of the figures for publication the authors inadvertently inserted Figure 1 for Fig 3. Please see the correct Fig 3 here.

**Table 2:** Features of tagged members of the *pir*, *fam-a* and *fam-b* multigene families.

Name tagged protein	Fluorescent tag	Mutant name	RMgmDB ID^1^	BLOOD				LIVER
				expression and localisation	% of clones fluorescent	fluorescent before passage (%)	fluorescent after passage (%)	protein localisation
**Single gene-tagging mutants (multigene families)**								
Fam-a1	mCherry	1477cl3	690	Yes; RBC s	33% (n = 3)	80–90%	50–70%	Yes; PV
Fam-a1	GFP	1941	1283	Yes; RBC s	N.A.	25–30%	30%	No
Fam-a2	mCherry	1448cl2	693	Yes; RBC c pa	60% (n = 5)	99%	85–100%	Yes; PV
Fam-b1	mCherry	1599cl4	699	Yes; RBC c pa	66% (n = 3)	5–15%	5%	Yes; PV
Fam-b2	mCherry	1731cl4	700	Yes; RBC c pa	100% (n = 5)	65%	50–70%	Yes; PV
Fam-b2	GFP	1942	1282	Yes; RBC c pa	N.A.	50–75%	N.D.	N.D
PIR1	mCherry	1531cl3	695	Yes; RBC c pa	100% (n = 5)	15%	60–70%	Yes; PV
PIR1	mCherry	1944cl1	1281	Yes; RBC c pa	66% (n = 3)	10–20%	N.D.	N.D.
PIR2	GFP	603cl3	696	Yes; RBC c pa	75% (n = 4)	5–10%	5%	No
PIR3	mCherry	1918cl4	697	Yes; RBC c pa	25% (n = 4)	1%-5%	N.D.	No
PIR4	mCherry	2450	1233	Yes; RBC c pa	N.A.	0.1–2%	N.D.	No
PIR5	mCherry	2448cl1	1234	Yes; RBC c pa	100% (n = 3)	25–50%	N.D.	N.D.
PIR6	mCherry	1892	698	Yes; RBC c pa	N.A.	<0.1%	N.D.	N.D.
PIR7	mCherry	2211	1235	Yes; RBC c pa	N.A.	<0.1%	N.D.	N.D.
PIR8	mCherry	2312, 2313	1236	Yes; RBC c pa	N.A.	50–60%	30–60%	yes, parasite cyt
**Double gene-tagging mutants (multigene families)**								
Fam-a2 Fam-a1 *Fam-a2/a1*	mCherry GFP mCherry&GFP	2010 (2011)	1244	Yes; RBC c pa Yes; RBC s	N.A.	70–80% 40–65% 40–55%	70–80% 45–50% 30–40%	Yes; PV (>90%) No
Fam-a2 Fam-a1 *Fam-a2/a1*	GFP mCherry GFP&mCherry	2504cl3	1245	Yes; RBC c pa Yes; RBC s	100% (n = 3)	80–90% 80–90% 80–85%	75–80% 60–65% 70–80%	Yes; PV (70–75%) Yes, PV (30–40%) 30–40%
Fam-b1 Fam-b2 *Fam-b1/b2*	mCherry GFP mCherry&GFP	2421(-2424)	1246	Yes; RBC c pa Yes; RBC c pa	N.A.	40–50% 40–45% 35–45%	30–50% 50–80% 20–40%	Yes; PV No
PIR1 PIR3 *PIR1/PIR3*	mCherry GFP mCherry&GFP	2020 (2021)	1247	Yes; RBC c pa Yes; RBC c pa	N.A.	35–45% 25–35% 20–30%	40–60% 1–20% 1–5%	Yes; PV No
**Single gene-tagging mutants (single copy genes)**								
IBIS1	GFP mcherry	2009 1940cl1	1237	Yes; RBC c pu	N.A. 100% (n = 1)	N.D >90%	N.D. N.D.	Yes; PV (>90%)
SMAC	mCherry	1565cl1	1238	Yes; RBC c pa	100% (n = 4)	>90%	N.D.	Yes; PV (>90%)

^1^ www.pberghei.eu

**Fig 3:**
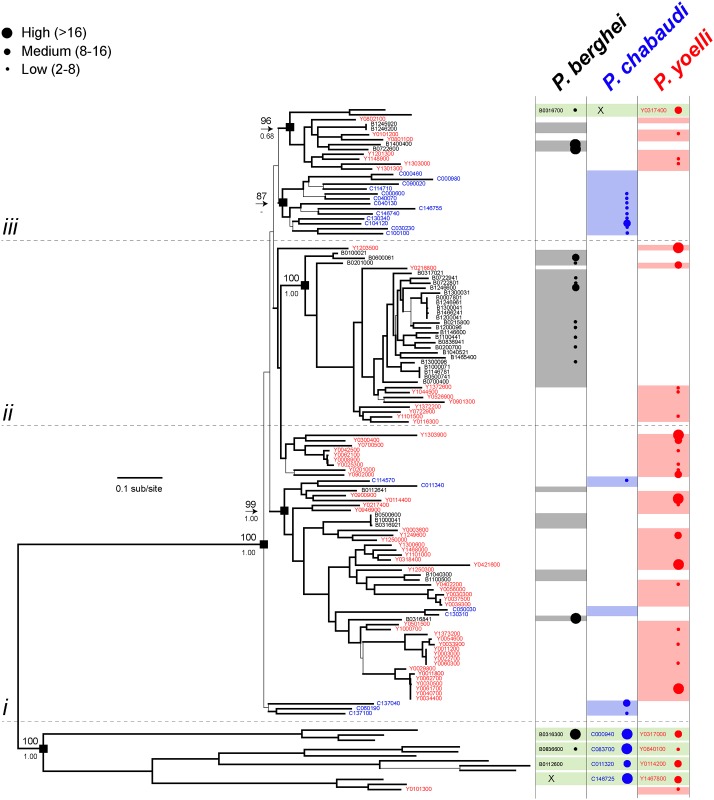
Maximum likelihood phylogeny of *fam-b* gene sequences from *Plasmodium* spp. The tree was estimated using RAxML and a GTR+Γ model. Branches subtended by nodes with >75 bootstrap support are shown in bold. Robust basal nodes are indicated by black squares with bootstrap proportions (above node) and Bayesian posterior probabilities (beneath node). At right, coloured blocks indicate the species to which a terminal node belongs. Clades of orthologs that display positional conservation are indicated with green blocks; where a sequence has been lost secondarily in one species, this is shown by an ‘X’. The phylogeny is subdivided into four sections: divergent genes included conserved loci, placed at the root of the tree (below line *i*); predominantly *P*. *yoelli* species-specific genes *P*. *berghei*- and *P*. *yoelli*-specific paralogs (between lines *i*, *ii* and *iii*); and predominantly *P*. *chabaudi* species-specific genes (above line *iii*). Transcription levels (shown as different coloured and sized circles) in blood stages are shown for individual genes based on RNAseq data (FPKM values) (from [33] and **S1** Table). Expression levels as shown by four different sized circles: Class 1 (smallest circle): 2-8x the threshold level; class 2: 8-16x the threshold; class 3 (largest circle): >16x the threshold.
