# Supplementary figures and images for: Variant Exported Blood-Stage Proteins Encoded by Plasmodium Multigene Families Are Expressed in Liver Stages Where They Are Exported into the Parasitophorous Vacuole
Source: PLoS Pathog. 2016 Nov 16;12(11):e1005917. doi: 10.1371/journal.ppat.1005917 (PMC5113031; doi:10.1371/journal.ppat.1005917)

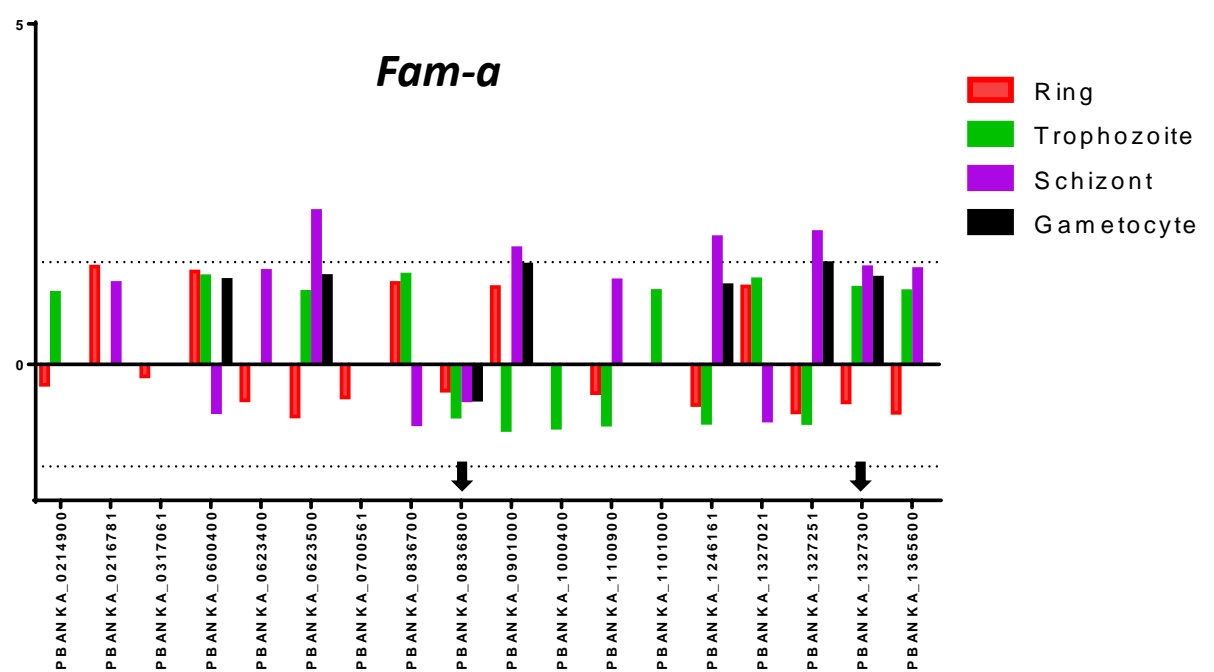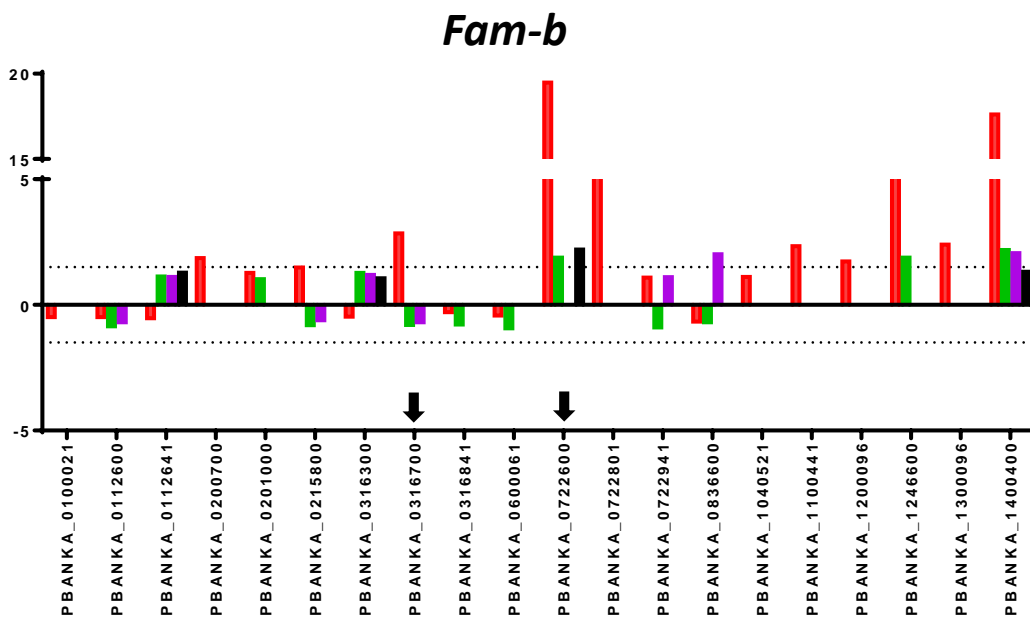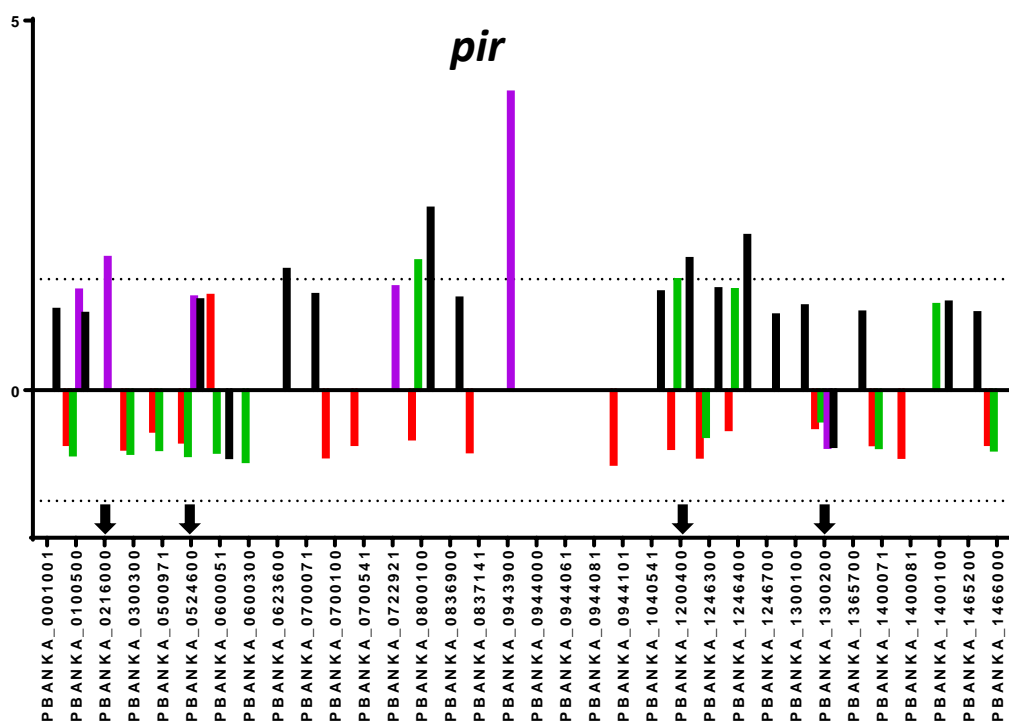

Supplement: S1 Fig — The fold up- or down-regulation of expressed genes (RNAseq FPKM values >21; S1 Table) is calculated by dividing the FPKM value observed for each gene for a particular blood stage by the FPKM value for the same gene and blood stage of the other line. Dotted lines show a 1.5 fold up- or down regulation. Arrows indicated genes selected for tagging. (PDF) [file ppat.1005917.s001.pdf]

A. PBANKA\_0519300::mCherry (line 2086)

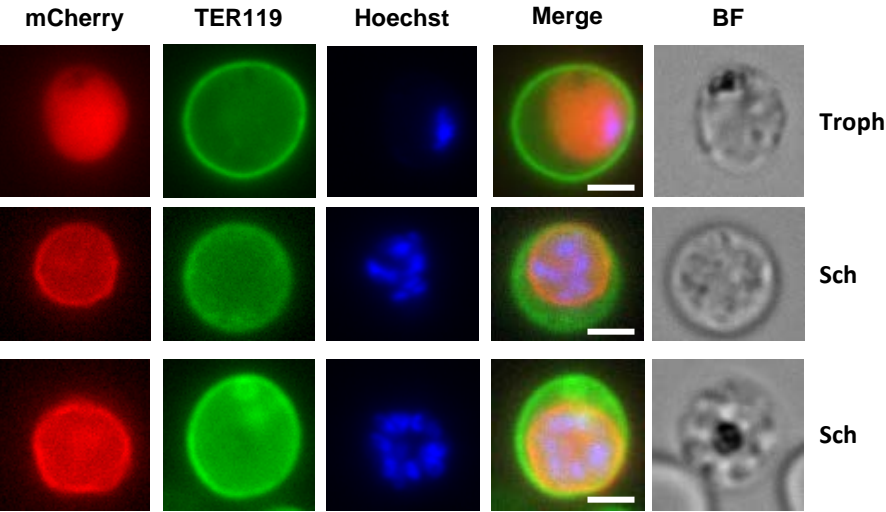

B. PBANKA\_1400600::mCherry (line 1915)

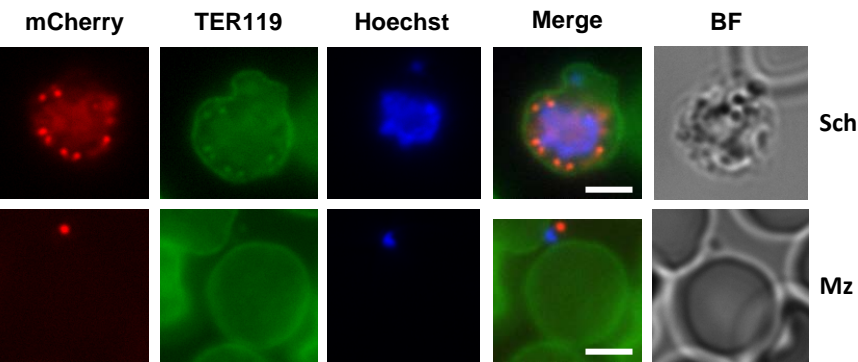

Supplement: S2 Fig — A. Location of mCherry-tagged PBANKA_0519300 (line 2086) in the cytoplasm of trophozoites and schizonts. B. Location of mCherry-tagged PBANKA_1400600 (line 1915) with an organellar (rhoptry‐like) localization in merozoites. The plasma membrane of the red blood cell is stained with TER119 antibodies (green) and parasite nuclei are stained with Hoechst. Troph: trophozoite; Sch: schizont and Mz: merozoroite. Scale bar: 2μm. (PDF) [file ppat.1005917.s002.pdf]

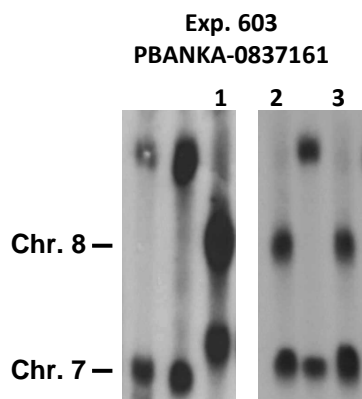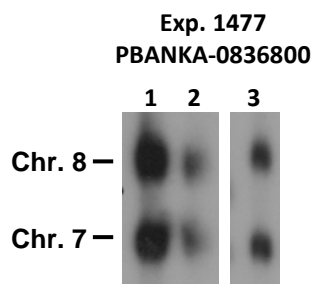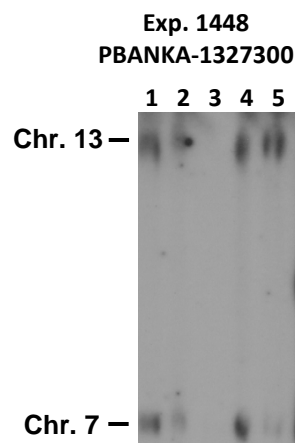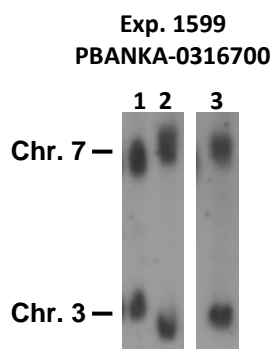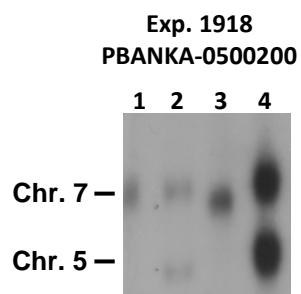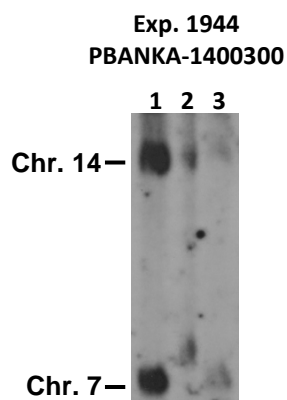

Supplement: S3 Fig — Separated chromosomes were hybridized with a probe recognizing the 3’UTR of the bifunctional dihydrofolate reductase-thymidylate synthase (dhfr/ts) gene of P. berghei. This probe recognises the endogenous 3’UTR of the dhfr/ts located on chromosome 7 and the 3’UTR of the integrated construct into the target gene for tagging with mCherry or GFP. (PDF) [file ppat.1005917.s003.pdf]

### A. Fam-a1::cherry

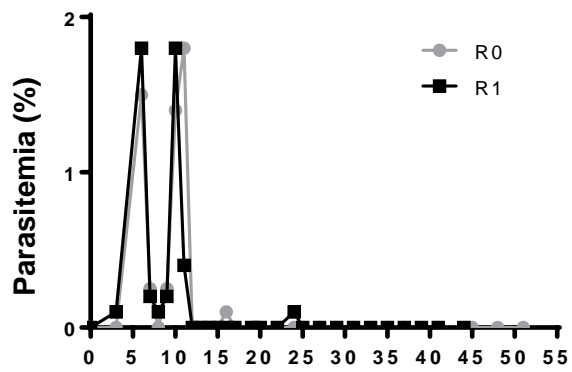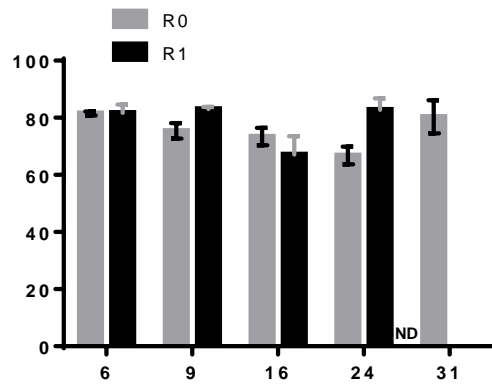

### B. Fam-b1::cherry and Fam-b2::cherry

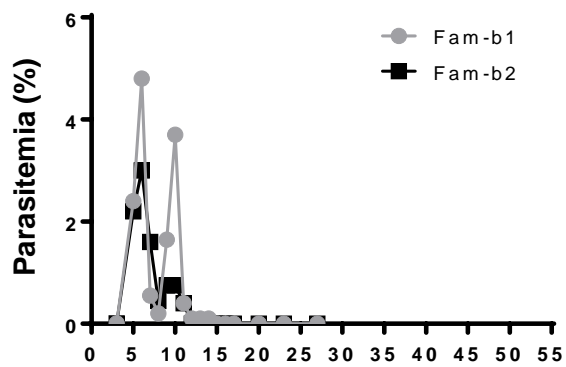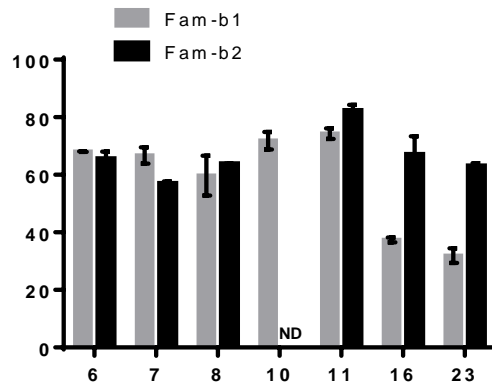

### C. PIR1::cherry

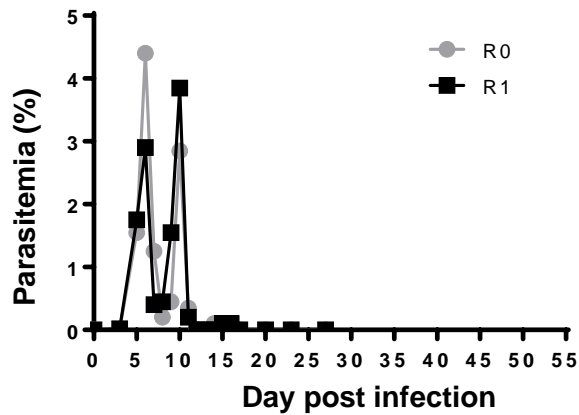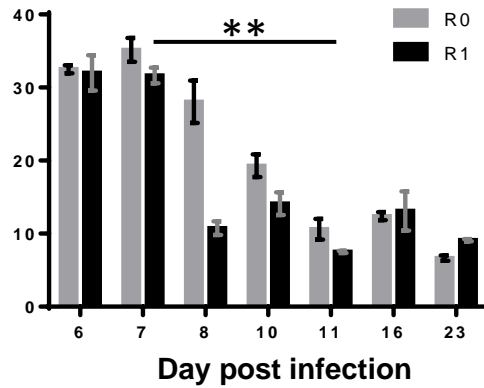

### D. wt-GFP-luc

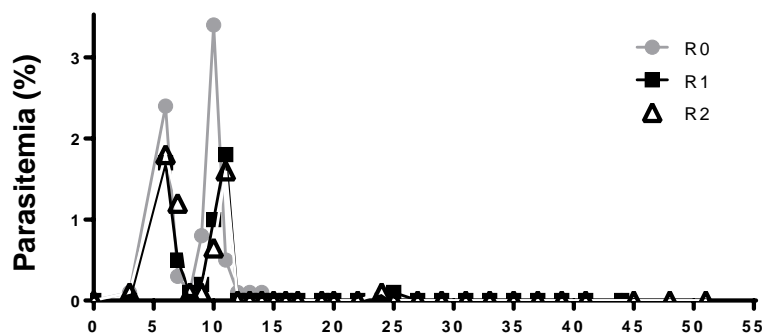

Supplement: S4 Fig — Percentage of fluorescent-positive schizonts (right panels) of cloned transgenic parasites expressing fluorescently tagged fam-a (A), fam-b (B) and pir (C) members during long-term infections in Brown Norway rats (2 rats per line R0 and R1 for Fam-a1 and PIR1; 1 rat for Fam-b1 and Fam-b2). In the left panels the course of parasitemia is shown in the rats. D. The course of parasitemia in rats infected with of a reference P. berghei ANKA line. **: p = 0.0062 (Two-way ANOVA). (PDF) [file ppat.1005917.s004.pdf]

A

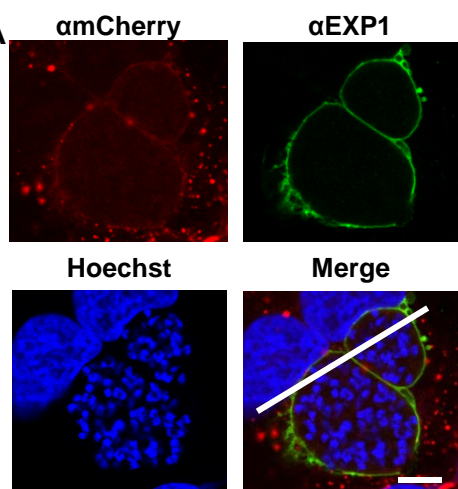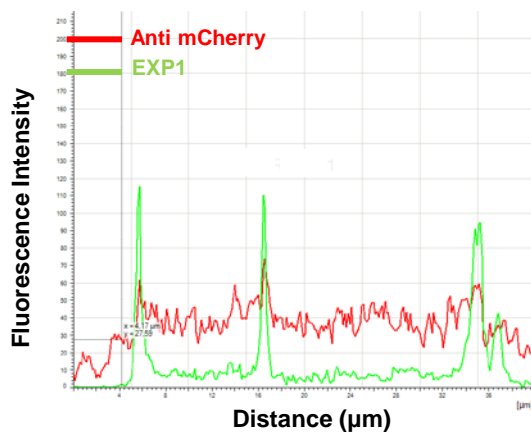

Fam-a1::cherry

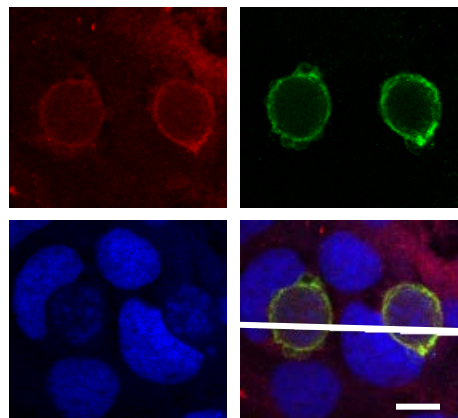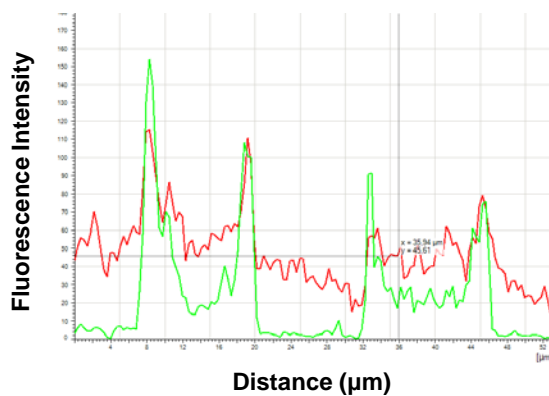

Fam-a2::cherry

B

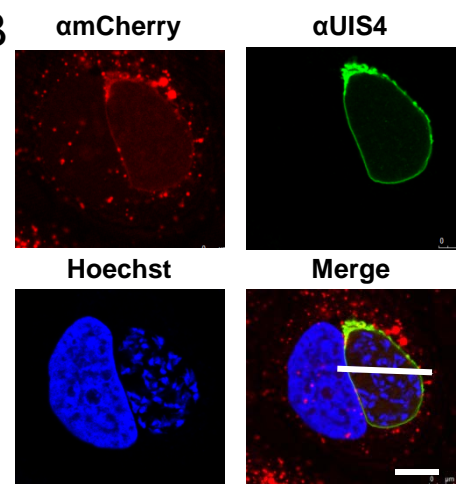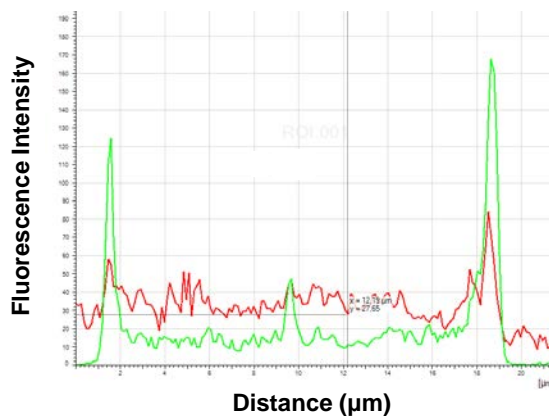

Fam-a1::cherry

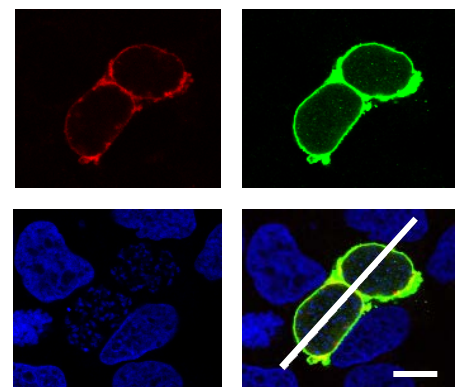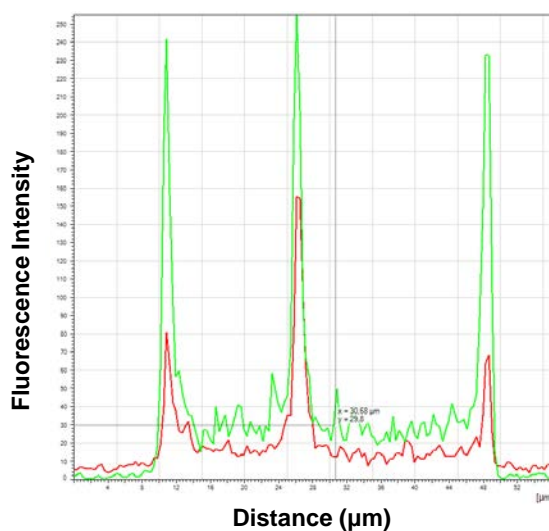

Fam-a2::cherry

Supplement: S5 Fig — Huh7 cells were infected with sporozoites of transgenic lines expressing either mCherry-tagged Fam-a1 or mCherry-tagged Fam-a2, fixed at 44 hpi and stained with antisera against two PVM-resident proteins (A. EXP1; B. IUS4; green) and with anti-mCherry antibodies (red). Fluorescence intensities for each fluorochrome were measured along the white line shown in the overlay image and plotted as distance versus intensity. Peaks of mCherry-staining overlap with both EXP1 and UIS4 staining. Nuclei are stained with Hoechst-33342 (blue). Scale bar: 2.5 μm, except for A lower panel, 10μm. (PDF) [file ppat.1005917.s005.pdf]

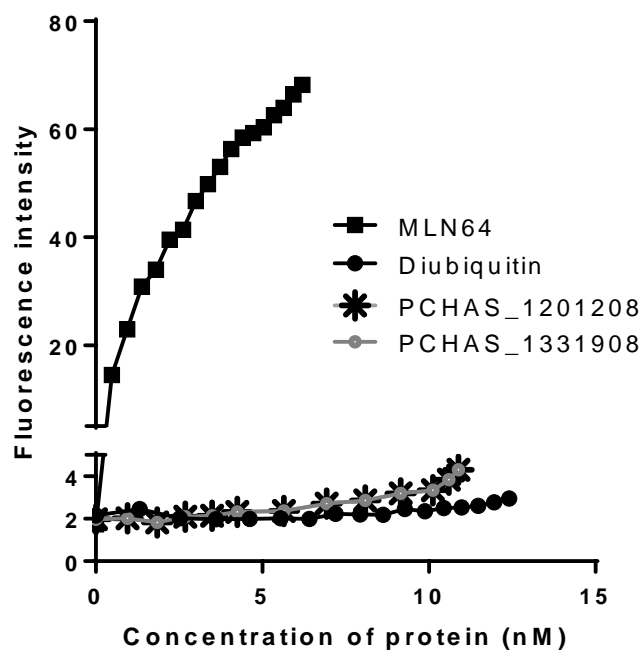

Supplement: S6 Fig — The binding of cholesterol by the recombinant Fam-A proteins PCHAS_1201200 and PCHAS_1331900 was tested by adding increasing amounts of protein to a solution containing 600 nM NBD-cholesterol. The emission of the fluorophore increases when it moves from the hydrophilic environment of the aqueous solvent to the hydrophobic environment of the binding pocket of the START. Hence an increase in amount of light emitted from the fluorophore indicates binding of the NBD-cholesterol to the START domain. In this case, no increase in emission was detected upon addition of the PCHAS_1201200, PCHAS_1331900 or the negative control, diubiquitin fused to a hexahistidine tag. Addition of the positive control protein MLN64 (also fused at its N terminus to a hexahistidine tag), lead to a steady, concentration-dependent increase in fluorescence emission, indicative of cholesterol binding. (PDF) [file ppat.1005917.s006.pdf]
